# Light-Driven Carbon Nitride Panel Technologies for Emerging Photocatalytic Applications

**DOI:** 10.1021/jacsau.6c00014

**Published:** 2026-03-30

**Authors:** Venugopala Rao Battula, Gabriel Mark, Michael Volokh, Menny Shalom

**Affiliations:** †Department of Chemistry and ‡Ilse Katz Institute for Nanoscale Science and Technology, 26732 Ben-Gurion University of the Negev, Beer-Sheva 8410501, Israel

**Keywords:** carbon nitride, photocatalysis, photocatalytic panel, thin film, photoreactor

## Abstract

Heterogeneous photocatalysis offers a sustainable alternative to many energy-intensive industrial processes; however, its scalability remains limited because common photoreactor designs rely on powder-based photocatalysts. This perspective explores the transition from traditional batch powder photocatalysis to scalable continuous-flow photocatalytic panels, with a focus on polymeric carbon nitride (CN) materials. Although CN has beneficial properties, such as ease of synthesis and stability, its use has been mainly limited to suspended powder systems for hydrogen production, hydrogen peroxide formation, CO_2_ reduction, and organic transformations. We review recent advancements in the development of CN-based photocatalytic panels (PCPs), highlighting scalable synthesis methods, including *in situ* growth techniques that enable direct polymerization onto substrates. The perspective covers photocatalytic system designs, PCP synthesis methods, structural characterization techniques, and applications in both batch and flow reactors. We highlight key challenges in transitioning from lab-scale to commercial-scale production and propose future research directions for CN photocatalytic panels, including learning opportunities from powder photocatalysis and photoelectrochemical systems. This analysis aims to connect laboratory demonstrations with future PCP implementation in industry.

## Graphical abstract

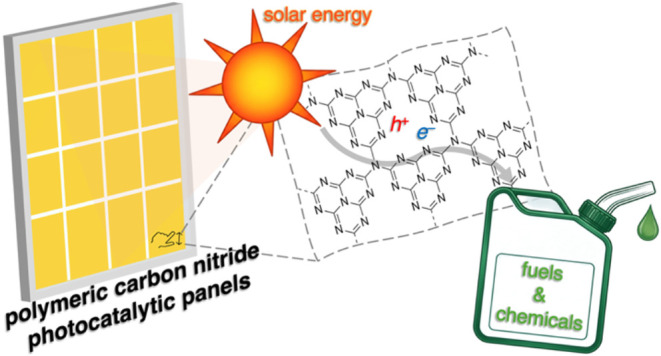

## 1 Introduction

The rapid progress in heterogeneous photocatalysis offers a promising, sustainable alternative to various energy-intensive industrial processes, including H_2_, H_2_O_2_, and fine chemical production.^1,2^ Currently, the photocatalysis field relies mainly on powder photocatalysts, which offer simplicity but limit the scalability and commercialization of the processes. Recently, a step forward in commercialization was demonstrated by using photocatalytic panels (PCPs) for solar fuel production.^3,4^

Turning the well-established lab-scale photocatalytic batch reactions—which involve a continuously stirred mixture of a heterogeneous photocatalyst and reactants—into a prototype continuous-flow device—which involves reactant(s) flow through a photocatalyst coated on a substrate—requires not only an additional performance optimization but also scalable PCP synthesis protocols. From a commercial perspective, the expected bottleneck is developing a scalable synthesis of large-sized PCPs.

The polymeric carbon nitride family of materials (CN) is a promising heterogeneous photocatalyst due to its ease of synthesis, tunable photophysical properties, and high stability. For more detailed information on the synthesis, characterization, properties, and applications of CN, the readers can refer to published detailed reviews.^5–9^ Despite CN’s many merits and broad demonstrated applications, it is primarily applied as a suspended powder for photocatalytic applications, *e.g.*, hydrogen evolution reaction (HER),^10^ oxygen reduction to hydrogen peroxide,^11^ CO_2_ reduction,^12^ and organic transformations.^13^ Only limited studies show CN films as scalable PCPs.^3,14–19^

This perspective highlights recent progress in PCPs and then delves into the synthesis and applications of CN-based PCPs, suggesting possible research directions and prospective applications. We summarize the latest lab-scale technologies for CN panel synthesis and their photocatalytic applications in both batch and flow reactors. Thus, we organize the perspective as follows: Section 2 discusses prominent photocatalytic architectures and notable applications. Section 3 introduces CN materials, describing the latest film and panel synthesis methods, emphasizing *in situ* growth (polymerization directly over substrates). Section 4 focuses on the influence of precursors/substrates on the structural properties of CN PCPs. The photocatalytic applications of these panels are discussed in Section 5, which is divided into subsections for each reaction type. In Section 6, future development avenues for CN-based PCPs are detailed, emphasizing the expected knowledge transfer from photocatalytic powders and photoelectrochemical electrodes, followed by general conclusions (Section 7) and a summary of the main challenges and development perspectives in the CN-based PCP realm in Section 8.

## 2 Photocatalytic Panels (PCPs)

Over the last few decades, photocatalysts have been developed and extensively studied for diverse applications, including solar fuel production, organic transformations, and water purification. Selected notable configurations are outlined in Figure 1. Prominent batch reactions rely on one of the following: (a) powder suspension, (b) self-standing^20^ or (c) floating^21^ hydrogel, (d) floating membrane,^22^ (e) thin film on the inner walls of a container,^23^ (f) slurry bubble,^24^ and (g) immersed coated substrate.^14^ Continuous-flow configurations may comprise: (h) fixed-bed,^25^ (i) thin film,^26^ (j) foam,^27^ (k) panel,^3^ (l) membrane,^28^ and (m) stirred slurry cascade.^29^ Additionally, photocatalysts were also immobilized on 3D printed supports,^30^ carbon fibers,^31^ and polyurethane foams^32^ in batch configurations and very recently on plastic materials as floating composites.^33^

**Figure 1:**
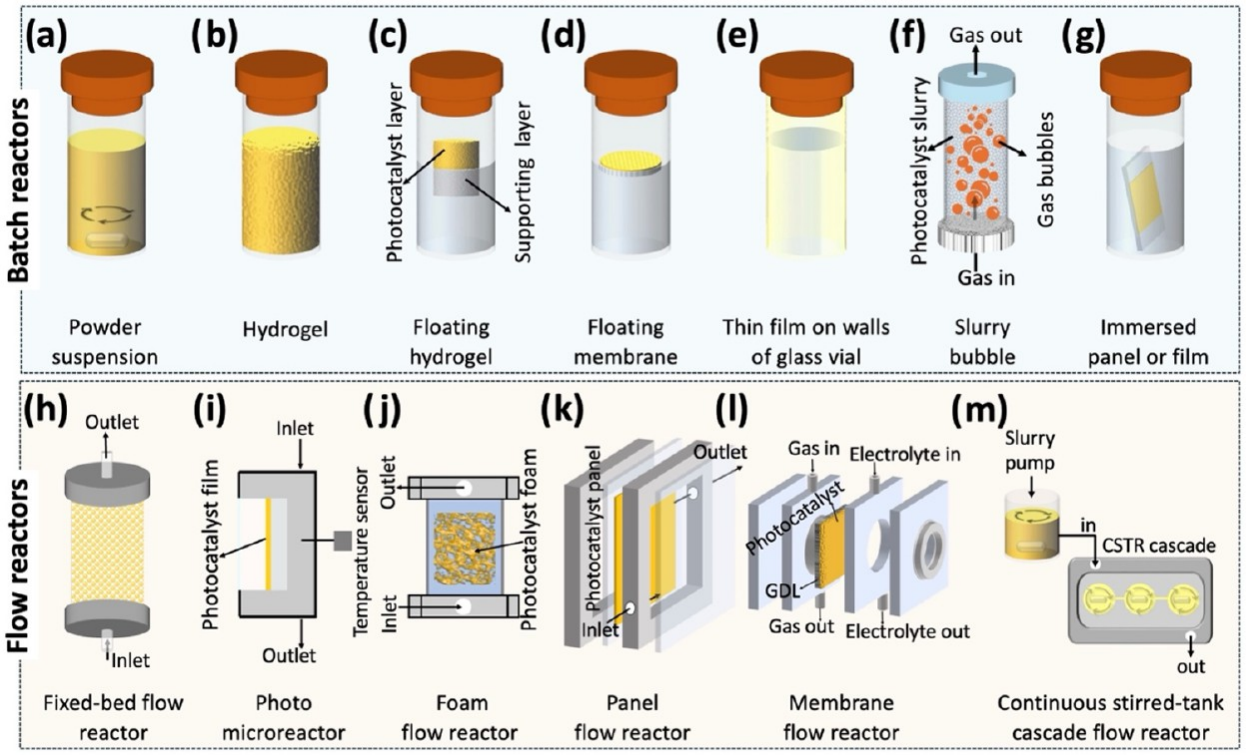
Photocatalyst implementation in photoreactor configurations. Batch mode: (a) Powder suspension, (b) self-standing hydrogel, (c) floating hydrogel, or (d) membrane, (e) thin film-coated walls, (f) slurry bubble configuration, (g) immersed PCP. Continuous-flow operation: (h) fixed-bed, (i) thin film, (j) foam, (k) panel, or (k) membrane, and continuous stirred-tank reactor (CSTR) cascade.

A photocatalytic panel is typically a substrate coated with a photocatalyst material that utilizes light to drive chemical reactions. From a commercial perspective, PCP configurations attract special interest due to the following key aspects: (1) Better light absorption compared to suspended powders (powders scatter more light and hinder its effective utilization) or compared to photocatalyst embedded in a hydrogel or foam (since light penetration into the material’s bulk is often challenging). (2) Potential scale-up to large sizes (on the m^2^ scale) without compromising light absorption. (3) Possible recyclability and easier product separation in an inherently heterogeneous configuration. (4) Small reaction volumes are viable, if required.

For panel fabrication, photocatalysts are commonly immobilized on solid substrates, such as stainless-steel (SS)^3,34^ and metal-oxides—glass^14^ or fluorine-doped tin oxide (FTO)-coated glass.^16^ The ideal substrate depends on the configuration. Still, glass is most used due to its transparency (crucial when the irradiation direction is limited),^14^ low cost, abundance, and high thermal and chemical stability. The two main PCP operational configurations are a batch setup, characterized by panel immersion (Figure 1g), for small-scale testing, or serving as the wall of a photoreactor (akin to Figure 1k, but static, without flow); and a continuous-flow setup (Figure 1k) for prototyping scale-up, under dynamic flow or recirculation.

### 2.1 Water Purification and Environmental Remediation

The thin-film configuration for water purification and environmental pollution remediation is well established and has been thoroughly reviewed in recent literature.^35–37^ Thongsuriwong et al. prepared ZnO thin films by the sol–gel dip-coating method for antibacterial activity and photocatalytic methylene blue degradation (PCMBD), a prominent model dye degradation experiment.^38^ Pham et al. prepared 2 × 2 cm^2^ Cu-doped TiO_2_/reduced graphene oxide thin films by spray coating for PCMBD.^39^ Poongodi et al. prepared 1 cm^2^ Nd-doped ZnO thin films *via* sol–gel spin-coating for PCMBD and antibacterial applications.^40^ Mohite et al. prepared WO_3_ thin films using spray pyrolysis for photoelectrochemical benzoic acid degradation.^41^ Additional PCMBD examples include top-down sol–gel deposition of TiO_2_ and N-doped TiO_2_ films on quartz (Lee et al.)^42^ and WO_3_ films on W-foil (Hu et al., who utilized a hydrothermal approach).^43^

### 2.2 Photoreactors

Fouad et al. immobilized W–TiO_2_ on 2 mm-thick SS plates (20 × 20 cm^2^) using polysiloxane for sulfamethazine degradation in a water-sliding photoreactor.^44^ Similarly, Ru/WO_3_/ZrO_2_ was immobilized on SS plates and used for disinfection of raw surface waters.^45^ Samy et al. coated graphene nanoplatelets/ZrV_2_O_7_ ^46^ or MIL-53­(Al)/ZnO^47^ on glass plates (5 × 7 or 5 × 2 cm^2^) using polysiloxane for the degradation of chlorpyrifos or trimethoprim, respectively, in a two-chamber photoreactor. For detailed information on the types of photoreactors used for water and air purification, we refer the reader to a review by Alalm et al.^48^

### 2.3 CO_2_ Reduction

Lee et al. prepared NiS-sensitized TiO_2_ films for photocatalytic CO_2_ reduction to CH_4_.^49^ Fereidooni et al. used commercial indium-doped tin oxide (ITO) films over glass or over flexible polyethylene terephthalate (PET) (4 × 7.5 cm^2^) for CO_2_ reduction to CO and CH_4_.^50^

### 2.4 Water-Splitting

PCPs for water splitting have attracted significant interest during the past decade.^51^ In 2014, Domen’s group (Xiong et al.) pioneered the fabrication of 5 × 5 cm^2^ PCPs for overall water-splitting, which utilize Rh_2–*y*_Cr_*y*_O_3_/(Ga_1–*x*_Zn_*x*_)­(N_1–*x*_O_*x*_), prepared *via* drop casting.^52^ In the following year, the same group (Wang et al.) fabricated a 3 × 3 cm^2^ SrTiO_3_:La,Rh/Au/BiVO_4_ plate using the particle-transfer method for Z-scheme water-splitting.^53^ This method allowed Domen and co-workers to develop additional photocatalyst sheets.^54–59^ Later, they also used screen-printing and fabricated 30 × 30 cm^2^ printable photocatalyst sheets.^60^ In 2015, Schröder et al. developed a Pt@mp-CN panel (nine panels of 30 × 28 × 0.1 cm^3^, arranged into a large panel) using a simple drop-casting method for large-scale solar hydrogen production using a sacrificial electron donor (Figure 2a).^3^ In 2018, Domen’s group (Goto et al.) developed a 1 × 1 m^2^ RhCrO_*x*_/SrTiO_3_–Al panel (nine 33 × 33 cm^2^ panels arrayed together) for solar hydrogen production from water (Figure 2b).^61^ We refer the reader to review papers by Wang and Domen^62^ and Ravi and Noh^51^ for further details on particulate photocatalysts and scalable thin film approaches for panel/sheet fabrication. Nishiyama et al. impressively scaled up their previous 1 m^2^ demonstration to the 100 m^2^ scale for several months under real-world conditions (Figure 2d).^4^ Recently, Kudo’s group (Nagatsuka et al.) fabricated metal-sulfide-based Z-scheme photocatalyst sheets using drop-casting for visible-light-driven water-splitting (Figure 2e).^63^

**Figure 2:**
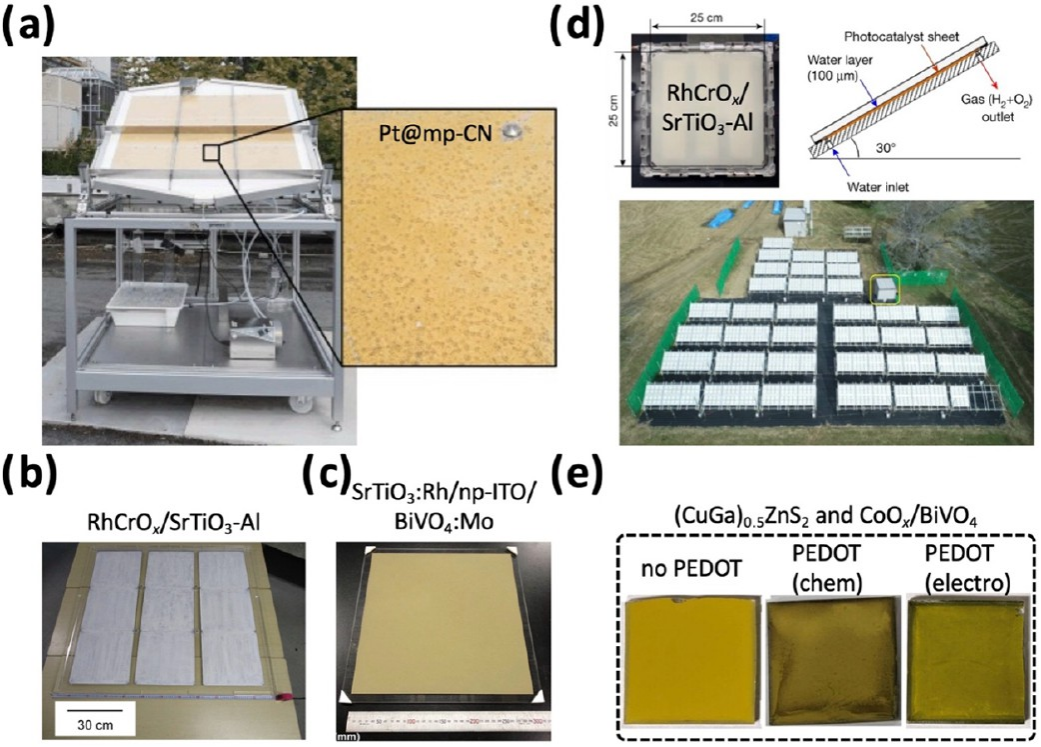
Photocatalyst panels. (a) Pt@mp-CN for large-scale solar hydrogen production. Reproduced with permission from ref 3. Copyright 2015 WILEY-VCH Verlag GmbH & Co. KGaA, Weinheim. (b) RhCrO_*x*_/SrTiO_3_–Al for water-splitting. Reproduced with permission from ref 61. Copyright 2017 Elsevier Inc. (c) Screen-printed 30 × 30 cm^2^ SrTiO_3_:Rh/np-ITO/BiVO_4_:Mo sheet. Reproduced with permission from ref 60. Copyright 2018 Elsevier Inc. (d) 100 m^2^ solar hydrogen production demonstration using RhCrO_*x*_/SrTiO_3_–Al. Reproduced with permission from ref 4. Copyright 2021 The Author(s), under exclusive license to Springer Nature Limited. and (e) (CuGa)_0.5_ZnS_2_ and CoO_*x*_/BiVO_4_-based Z-scheme sheets with PEDOT modifications for water-splitting. Reproduced from ref 63. Available under a CC-BY 4.0 license. Copyright 2025 The Authors.

### 2.5 Prospects of Polymeric Carbon Nitride (CN) PCPs

CN PCPs offer several advantages over traditional metal-oxide (MO) PCPs, including visible-light response with a tunable optical bandgap and nontoxic, metal-free, earth-abundant precursors. CN exhibits chemical stability on par with the stable MOs, making CN PCPs suitable for the majority of photocatalytic applications that require a wide pH range. Due to a more negative valence-band position, CN PCPs are effective for selective oxidation (biomass or organic oxidation), whereas using MOs often results in deep oxidation. Owing to their polymeric nature and ease of synthesis, CNs’ photophysical properties are readily tuned (*e.g.*, by doping, copolymerization, or chemical modification). The theoretical maximum solar-to-hydrogen (STH) conversion efficiency of CN is about 5%, which is higher than that of SrTiO_3_ (≈1.5%),^64^ a well-explored MO-based photocatalyst in a panel configuration for overall water-splitting, further highlighting the potential of CN-based PCPs.

Despite the listed merits of CN PCPs, their utilization is still in its infancy. The low conductivity and high charge recombination must be addressed by leveraging CN’s porosity to match the photocatalytic efficiencies of existing PCPs. Though CN as a powder is a verified chemically robust photocatalytic platform, it has yet to be fully implemented in PCPs, where substrate–CN interactions and its robustness must be established before large-scale synthesis and successful long-term operation. Section 3 introduces synthetic approaches to achieve CN films over substrates, followed by a detailed discussion of the properties ensuing from the synthetic methodology (Section 4).

## 3 CN Panel Synthesis Methods

Carbon nitrides based on repeating units of heptazine (tri*s*-triazine) are commonly found as either the “melon” polymorph, as the ideal, fully condensed, graphitic C_3_N_4_ (g-C_3_N_4_), or as their mixture. Since it is hard to distinguish between them experimentally, we refer to these polymer configurations simply as CN. CN is a 2D metal-free organic semiconductor composed of carbon and nitrogen in its backbone with residual hydrogen atoms (NH and NH_2_ groups) and exhibits high chemical stability and interesting optical properties with a band gap of ca. 2.7 eV.^8^ Figure 3 represents a general scheme showing the (multistep) formation of melon and g-C_3_N_4_ from melamine.

**Figure 3:**
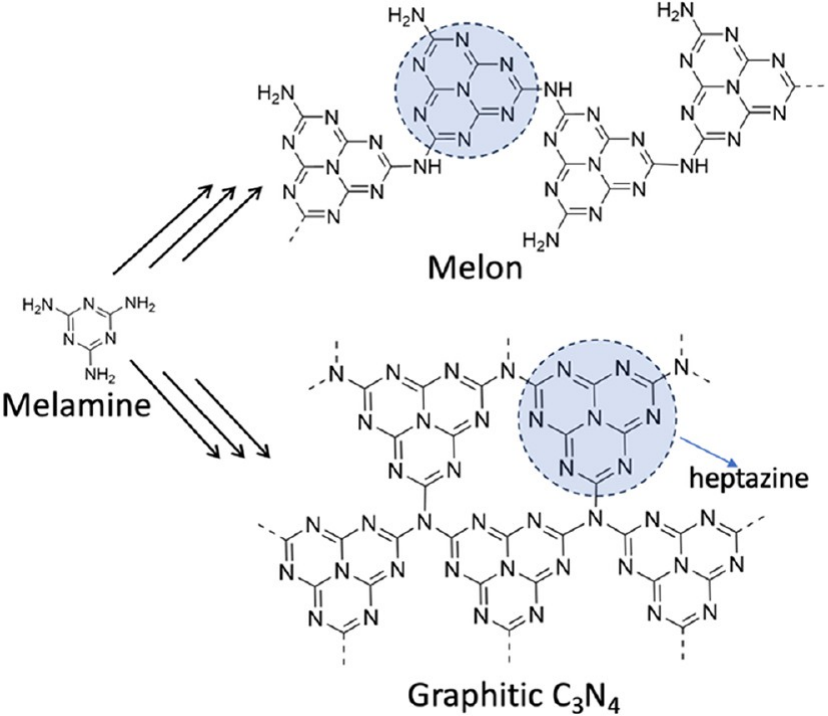
Synthetic scheme of polymeric carbon nitrides from a melamine precursor. The blue-highlighted unit is heptazine.

In their 2009 pioneering report, Wang et al. explored CN as a visible-light-active photocatalyst (in powder form) for hydrogen production for the first time.^10^ Since then, CN-based materials have been extensively studied for visible-light-driven photocatalytic applications such as hydrogen peroxide production, water purification, CO_2_ reduction, and water-splitting. CNs are easily synthesized through the thermal treatment of various C- and N-containing precursors, allowing the formation of the melamine monomer at high temperatures. It then undergoes a condensation reaction, liberating NH_3_(g), and forms in-plane aromatic heptazine units with dangling amine groups, which are stacked together by van der Waals interactions. For more detailed information on different types of CNs and related materials, their properties, and photocatalytic applications, we refer the readers to some excellent reviews.^6,7,65,66^

A typical PCP consists of a thin CN layer (ordinarily up to a thickness of tens of μm), grown, deposited, or immobilized on a flat solid surface. In a review from our group (Barrio et al.),^9^ we briefly summarized prominent CN deposition techniques. We have found that a key factor in improving film adhesion and growth is the presence of precursor vapors during polymerization, as detailed in our perspective (Volokh and Shalom)^67^ regarding photoelectrochemical (PEC) applications.

The direct growth methods of CN include gas-phase and liquid-phase syntheses. In the gas-phase synthesis, the vapor of CN precursors condenses on the substrate surface and polymerizes into the sought-after CN.^68–71^ In the liquid-phase, the substrate is directly immersed in liquid media (molten CN precursors)^72^ or in solvothermal solutions.^73^ Furthermore, Jia et al. have dedicatedly summarized the recent progress in the fabrication of CN thin films on substrates or as free-standing membranes, for various applications.^74^ In their review, the authors extensively discussed the existing thin film synthesis methods and classified them into two types: postprocessing and direct growth methods. Since these summaries are available, we focus on recent scalable CN panel preparation methods (Figure 4), which can be dedicatedly used for photocatalytic reduction and oxidation reactions—in both batch (Figure 1g) and continuous-flow configurations (Figure 1k). Adapting Jia et al.’s classification, we divide PCP preparation schemes into either the deposition of a thin layer of already-synthesized CN (*i.e.*, postprocessing) or the *in situ* polymerization of precursors over the solid substrate (*i.e.*, direct growth).

**Figure 4:**
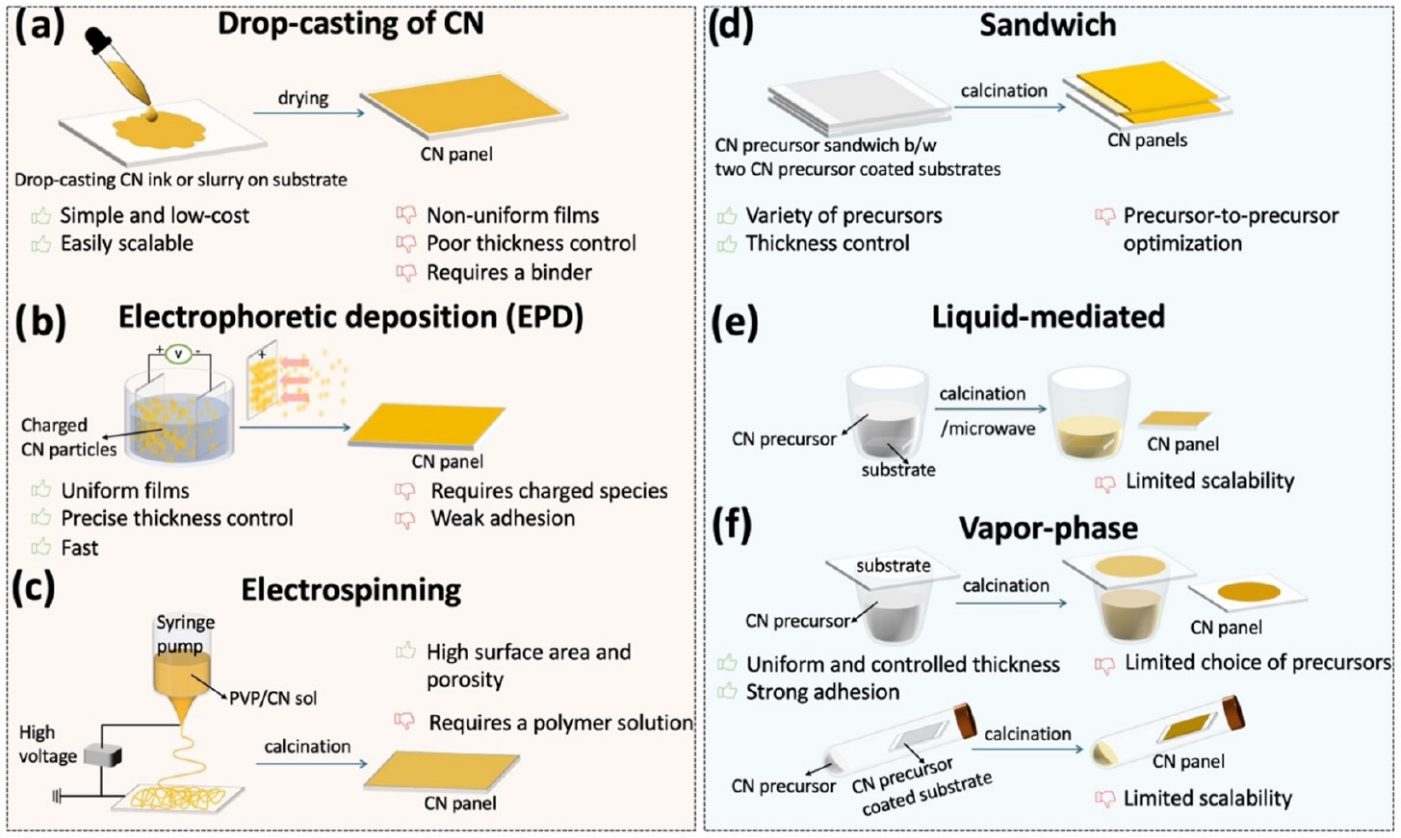
CN panel synthesis *via* (a–c) postprocessing and (d–f) direct growth methods. Postprocessing involves: (a) drop-casting a CN ink or slurry (typically, a synthesized CN powder and a binder) onto a target substrate, followed by drying; (b) Electrophoretic deposition of charged CN particles onto a conductive substrate;^75^ (c) electrospinning of a CN–polymer solution onto a conductive substrate under high voltage, followed by calcination.^76^ The direct growth employs an *in situ* thermal polymerization: (d) The “sandwich” method, where CN precursor(s) are sandwiched between two glass substrates or two CN precursor-coated glass substrates, followed by calcination to yield CN panels or thicker CN panels, respectively;^15^ (e) liquid-mediated method, where substrate buried in CN precursor, followed by calcination or microwave heating to yield CN panels;^72^ (f) vapor-phase method shows a thermal vapor condensation (TVC) method, where CN precursors are placed at the bottom of a crucible covered by glass substrates, followed by calcination to yield CN panels;^68^ and a vapor deposition polymerization (VDP) method, where both CN precursor and substrate or CN precursor-coated substrate are placed in a tube, followed by calcination to yield CN panels or thicker CN panels, respectively.^77^

### 3.1 Postprocessing: Deposition of Presynthesized CN

Depositing CN powders synthesized before the deposition step is one of the most convenient and well-established methods, even for large PCPs. This approach divides the synthetic process into two steps. The first step involves synthesizing a CN or its derivative in powder form, and the second step consists of coating the powder onto a substrate. Importantly, CNs can be readily synthesized in large quantities by thermal polymerization of precursors such as urea, melamine, or Dicyandiamide under an inert atmosphere.^9^

#### 3.1.1 Drop-Casting

The obtained CN can be dispersed to form a CN slurry or ink, which is then coated onto a substrate to form a CN panel. This method often requires a binder material (for example, Nafion) to improve CN adhesion to the surface. The main merit of the described approach is its simplicity and its potential scalability with commercial spray or spin coaters.

In 2015, Schröder et al. immobilized mesoporous CN (mp-CN) that contains Pt cocatalyst (Pt@mp-CN) onto SS (3.5 × 3.5 × 0.25 cm^3^ for lab-scale and 30 × 28 × 0.1 cm^3^ for large-scale). The large-scale reactor was assembled using nine such Pt@mp-CN-coated SS plates, with a total of 13.3 g of Pt@mp-CN deposited.^3^

In 2021, Uekert et al. prepared scalable CN_*x*_|Ni_2_P panels using a similar drop-casting methodology.^14^ The authors tested a variety of immobilization procedures—testing different deposition methods (spin-coating and drop-casting), solvents (H_2_O and ethanol (EtOH)), binders (poly­(ethylene glycol), Nafion, and SiO_2_ nanoparticles or microparticles), substrates (flat and frosted glass), and annealing temperatures (80–450 °C). A low-temperature and straightforward procedure was preferred for its performance after comparing photocatalytic activity and stability (neutral and alkaline conditions). The number deposition layers number (2–12) controlled the photocatalyst loading (0.64–3.84 mg_CN*x*_ cm^–2^).

In 2025, Rahaman et al. from the same group prepared a CN_*x*_–ITO|FDH biohybrid photocatalyst sheet on FTO. They combined *a* photocatalyst (cyanamide-functionalized carbon nitride, CN_*x*_, from their earlier report)^78^ and a biocatalyst (a metal-dependent formate dehydrogenase enzyme, FDH). Tin-doped indium oxide (ITO) nanoparticle powders were blended in various ratios and served as both an electron conduit and a support for FDH. Before the photocatalytic reaction, the FDH was also drop-cast onto the CN_*x*_–ITO 0.5 cm^2^ photoactive area. Similarly, 50 cm^2^ CN_*x*_–ITO sheets were prepared.^18^

Since CN powders do not readily dissolve or exfoliate in solvents, most achievable dispersions in solvents—such as water and alcohols—are, in fact, unstable suspensions. Therefore, after casting and drying, the CN layer would peel off the substrate unless a binder is added to the dispersion. Typical organic binders, such as Nafion, entail the functionality that provides adhesion to CN. When these binder-containing panels are applied for organic transformations, especially where CN generates reactive oxygen species (ROS) or H_2_O_2_, the binder’s carbonaceous backbone degrades, ultimately causing leaching of the CN layer. Moreover, the (partially reacted) binder’s presence may complicate product analysis or hamper purity. On the other hand, inorganic binders (SiO_2_ or ITO) can be used; however, the choice of binder should be studied on a case-by-case basis, as it can influence the reaction progress or pathway. Thus, we propose focusing research on binder-free CN PCPs.

#### 3.1.2 Electrophoretic Deposition (EPD)

In this method, first, CN powder is dispersed (for example, in toluene) to obtain a fine suspension. Next, a voltage is applied between the target substrate and the counter electrode. After deposition, an annealing step yields CN thin films over conductive substrates (FTO, carbon paper, Ni foam).^75^

In 2019, Rieß et al. prepared CN thin films on a SS plate by dispersing mesoporous CN and I_2_ (as a charging agent) in acetone.^79^ In 2023, Benedet et al. coated CN on Ni foam-supported MnO_2_ electrodes using a similar methodology.^80^

Though EPD is a convenient CN powder deposition method that allows thickness control, the large size and low solubility of CN particles result in a weak adhesion to the substrate.^79,81^

#### 3.1.3 Electrospinning

Electrospinning is a versatile technique that uses high voltage to generate polymer fibers from a solution of spinnable materials. It produces high-specific-surface-area (*S*_A_) and porous (nano)­fibers on the collector. For a nonspinnable material like CN, an additional polymeric binder, such as poly­(vinylpyrrolidone) (PVP), is required.^76^ For example, Wang et al. prepared C_PVP_/CN films on ITO.^76^ First, a CN nanosheet–PVP blend in water was prepared. After electrospinning, the films were calcined to obtain C_PVP_/CN over ITO.

However, when aiming for pristine CN films on transparent substrates, this technique is limited since electrospun fibers are randomly oriented, leading to nonuniform coverage and it requires an additional calcination step to remove the binder. While depositing presynthesized CN powders facilitates PCP fabrication and offers the advantage of using already-developed (tailored or modified) CN materials, challenges remain regarding binder requirements, adhesion, and uniformity. We therefore believe that the direct CN growth on substrates is a better alternative for scalable, practical photocatalysis.

### 3.2 Direct Growth: *In Situ* CN Film Condensation and Polymerization

In the *in situ* approach, the photoactive layer forms directly during the thermal polymerization and condensation of CN precursors over the substrate’s surface. It results in a binder-free, intimate contact between the growing CN and the substrate. The main obstacle to achieving controlled, substantial polymerization of precursors into the sought CN on the substrate’s surface is precursors’ volatility. We first discuss the “sandwich” approach, which confines the precursor intermediates near the surface. Next, we elaborate on liquid-phase synthesis, which mitigates precursor escape during polymerization. Then, we discuss dedicated vapor-phase methods that leverage the precursor’s volatility. Finally, in Section 3.2.4, we discuss prominent precursor-deposition modifications that affect successful direct growth.

#### 3.2.1 The “Sandwich” Method

In 2014, Shalom et al. reported a direct growth method for CN on several substrates, utilizing supramolecular assemblies (in this case, cyanuric acid–melamine, CM) as the CN precursor.^82^ Specifically, they ‘sandwiched’ a CM between two glass substrates, possibly precoated with another metal-oxide.

Interestingly, the interaction between the supramolecular precursor and the glass surface (free amine and hydroxy groups, respectively) yielded well-ordered rods. Using this approach, a ca. 60 nm thick CN layer over FTO was deposited and exhibited electrocatalytic activity. However, for photocatalytic applications, the layer’s light-harvesting capability must be significantly improved by increasing its thickness, which can be only partially addressed by the described “sandwich” method alone.

In 2018, our group proposed a general approach for the fabrication of large-scale porous CN films with controlled thickness and composition, utilizing the doctor-blade technique to deposit a thick layer of precursor(s) paste as a preliminary step.^83^ Specifically, a supramolecular assembly (*i.e.*, CM or barbituric acid-doped CM in that report) was dispersed into a paste after screening several dispersants. The optimal dispersion used a hydrogen-bonding solvent, ethylene glycol (EG). Coating this paste onto substrates (e.g., FTO) as the CN precursor yields uniform, porous CN layers after calcination, which exhibit good dye adsorption and degradation properties, as well as PEC activity.

Inspired by this approach, we envisioned that introducing an additional thin layer of a CN supramolecular precursor before “sandwiching” would increase the thickness of the ensuing CN layer.^15^ In this study, we first tested different CN precursors melamine (M) and melamine–cyanuric acid supramolecular assembly (MCA). Sandwiching the precursor between two glass substrates (6.7 × 6.7 cm^2^) and heating to 530 °C for 4 h, yielded two identical PCPs (CN_M_ and CN_MCA_; Figure 5). The CN_M_ panel exhibited a nonuniform CN coating, whereas CN_MCA_ showed uniformity. We ascribe the improved uniformity, in part, to the lower volatility of supramolecular aggregates. In a following study (Abed et al.),^17^ we adapted the described binder-free synthesis method (sandwich + doctor-blade) to sulfur-doped CN (CN-CMBT_1_) on glass and FTO (Figure 5).

**Figure 5:**
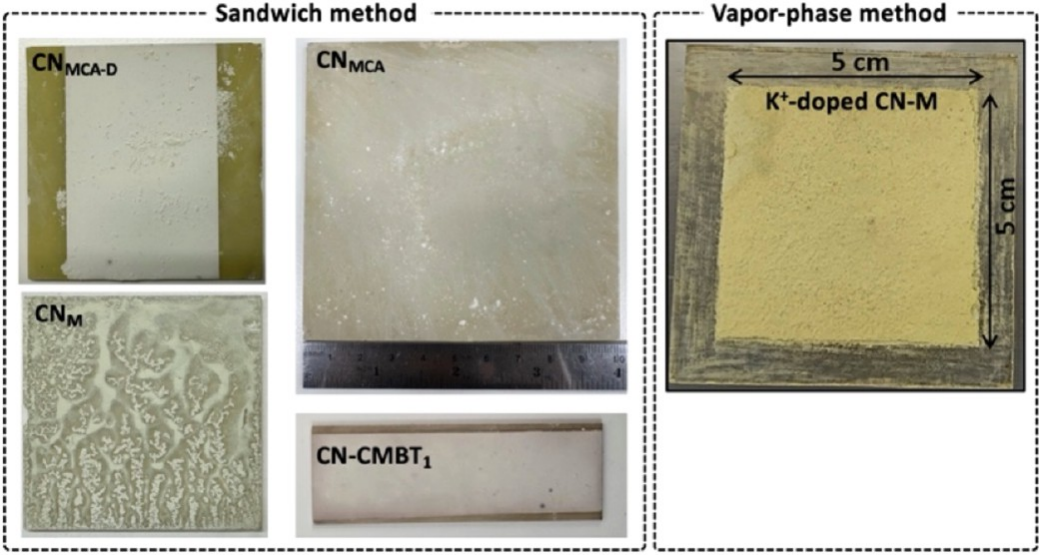
Images of CN PCPs synthesized using the *in situ* “sandwich” method from melamine (M) and melamine–cyanuric acid (MCA) supramolecular assemblies; CN_MCA–D_ adds a doctor-blading step to the “sandwich”; images of CN_MCA–D_, CN_MCA_, and CN_M_ were adapted from ref 15. Copyright 2024 American Chemical Society. CN-CMBT_1_ is an S-doped CN PCP synthesized from a bismuthiol-containing MCA precursor, as described in ref 17. Available under a CC BY-NC-ND 4.0 license. Copyright 2025 The Author(s). Vapor-phase synthesis from M over KCl-coated glass yields a potassium-doped CN PCP. Adapted from ref 19. Available under a CC BY-NC-ND 4.0 license. Copyright 2026 The Author(s). *Small* published by Wiley-VCH GmbH.

The scalability was further verified by synthesizing a 10 × 10 cm^2^ CN_MCA_ panel (Figure 5). In general, with a suitable-sized furnace, this method can be used to prepare even larger panels. However, as noted earlier, the thickness of the photoactive layer needed to be increased to improve light utilization. Therefore, we introduced an additional coating step (*via* doctor-blading) during CN_MCA_ panel synthesis. Briefly, an MCA in EG paste was doctor-bladed onto glass substrates. After drying, additional MCA powder was sandwiched between the two MCA-coated substrates. This combination enabled control over the thickness of the final CN coating up to ca. 68 μm.^15^

In a separate study, we adapted a rapid (minute-scale) CN photoanode preparation procedure at elevated temperatures^84^ to induce rapid polymerization of CN PCPs (Tashakory et al.).^16^ Briefly, a paste from a 3MelM precursor (3:1 molar ratio of melem–melamine supramolecular aggregate) dispersed in EG is doctor-bladed onto two FTO substrates. Melem was chosen because, unlike melamine, it does not sublime readily. The 3:1 molar ratio originated from earlier CN powder studies.^84,85^ Calcination at 680 °C for 10 or 20 min yielded panels labeled as CN-10 and CN-20, respectively. Similarly, CN-M10 and CN-M20 panels were prepared, with additional melem precursor between the 3MelM-coated substrates.

Despite its advantages, this approach often requires careful precursor optimization—for example, using the same mass of melamine or of the supramolecular MCA precursor yields different CN coatings (CN_M_ and CN_MCA_). Moreover, leftover CN powder between the substrates after calcination might stick to the CN layer and require additional treatment. Hence, this method should be optimized to test each modification and poses concerns for easy scale-up.

#### 3.2.2 Liquid-Mediated Methods

A prominent example is the immersion of a substrate into a precursor powder that melts upon heating (*e.g.*, a supramolecular aggregate of cyanuric acid–2,4-diamino-6-phenyl-1,3,5-triazine), resulting in a solid–liquid interface that is subjected to the polymerization temperature to form CN over the substrate.^72^ In fact, any method that deposits CN precursor(s) on a substrate, when heated, forms either a local gas-phase or liquid-phase that can condense into CN. A notable example from Zhang and co-workers is the deposition of cyanamide or Dicyandiamide by melting it and immersing an FTO substrate in the melt. Upon cooling, a solid precursor layer forms; then, the coated substrate is heated using microwave radiation to form CN.^86^ The exact state of the precursors or their intermediates is hard to probe during the reaction. However, since traditional thermal synthesis procedures occur at temperatures above 500 °C for several hours, polymerization mostly occurs at a solid/gas interface. We discuss typical gas-phase-only reactions in Section 3.2.3 and broaden the discussion to include precursor deposition alternatives and their combination with vapors in Section 3.2.4.

#### 3.2.3 Vapor-Phase Methods

In 2015, Bian et al. reported a thermal vapor condensation (TVC) method for growing CN on various substrates using the volatile melamine precursor.^68,71^ They placed the precursor in a ceramic crucible, covered it with a substrate, and upon heating, uniform CN films resulted. They observed that the thickness of the CN film varies with substrate type, increasing in the order ITO < silica < FTO < glass.^71^ Furthermore, the CN films grown on FTO, ITO, and silica showed similar structural and optical properties, whereas the CN film on glass showed similar properties to CN powder. Storage tests in air revealed that CN films on glass and ITO peeled off after 2 weeks, whereas those on FTO and silica remained stable for 6 months. In another study, they also tested other CN precursors (urea, thiourea, and Dicyandiamide) but observed a uniform film only with melamine.^68^ Additionally, their CN films were superior to those formed from CN powders by spin-coating and drop-casting in terms of surface coverage, roughness, and adhesion. The CN films on FTO survived ultrasonic treatment for 2 h.

Arazoe et al. introduced a variation they described as vapor-deposition polymerization (VDP), where an unsealed glass tube served as the reaction container.^77^ The CN precursor (melamine or guanidinium carbonate) was placed at the bottom, and a substrate was placed at the other side, near the tube’s mouth, covered with an aluminum cap.

In 2023, Chubenko et al. further developed a rapid chemical vapor deposition (CVD) method, analogous to the earlier TVC method, producing CN films (with stoichiometry close to ideal g-C_3_N_4_) on silicon and glass within minutes by directly placing the substrate and precursor in a preheated furnace.^87^ The listed approaches (and their variants, such as microcontact-printing-assisted deposition^88^) were demonstrated for CN electrodes or membranes, and are readily adaptable for CN PCP synthesis.

Recently, Garg et al. adapted vapor-phase melamine to synthesize 6.8 × 6.8 cm^2^ K^+^-doped CN PCP on a glass substrate, precoated with KCl (*i.e.*, underlayer).^19^ The KCl underlayer serves as both the K^+^ ion reservoir for doping and facilitates the CN growth, yielding K^+^-doped CN panels with cyanide defects. This modification demonstrates the feasibility of (i) metal-ion doping without the need for molten-salt media, (ii) indirect control over cyano-group defects, and (iii) thick (tens of μm) CN-based layers can be formed utilizing vapor-phase reaction, given an appropriate surface is present.

#### 3.2.4 Additional *In Situ* Growth Aspects

Since 2018, our group has explored methods for depositing precursors onto conductive substrates prior to their transformation into CN-based photoanodes without using the “sandwich” confinement. The doctor-blading technique^83^ is a scalable method that is suitable for deposition of various (combination of) molecular precursors and supramolecular aggregates. We also explored electrophoretic deposition,^81^ ultrasonic-spray coating,^89^ and dip-coating from supersaturated solutions.^90–92^ Each method has its specific advantages and disadvantages, but a common feature is that adding precursor vapors (VDP-style) improves adhesion and increases the thickness of the CN layer. A detailed discussion is out of scope since these electrodes were used for PEC-related applications.^5,67^ It is also possible to blend or alter the CN layer (with reduced graphene oxide,^93–95^ potassium,^96^ sodium and boron,^92^ or yttrium^97^) and form heterojunctions.^85,92,93,98^ Additional discussion of the relevance for PCPs appears in Section 6. Despite the extensive use of these methods for photoanode preparation, scaling up has been challenging thus far because substrates must be placed in covered glass vials or tubes, whereas this is not required with TVC and sandwich methods, as demonstrated in Figure 5.

### 3.3 Co-Catalyst Anchoring Strategies for *In Situ* Grown CN

CN requires cocatalysts for certain photocatalytic applications, such as oxygen evolution reaction (OER), hydrogen evolution reaction (HER), and nitrogen reduction, to provide redox-active sites and improve charge separation. Thus, we briefly discuss several promising reported cocatalyst anchoring strategies that are readily adoptable for CN PCPs.

#### 3.3.1 Photodeposition

This photoinduced, *in situ* technique deposits cocatalysts as nanoparticles directly on the photocatalyst’s surface, using the photogenerated electrons for the selective reduction of metal ions at the active sites. It is by far the most common technique for cocatalyst deposition in various photocatalytic applications (*e.g.*, HER). For example, Takata et al. photodeposited Rh/Cr_2_O_3_ and CoOOH cocatalysts onto SrTiO_3_:Al for overall water-splitting.^99^ We also photodeposited a Pt cocatalyst during photocatalytic HER over a CN_MCA_ panel.^15^

#### 3.3.2 EPD

EPD is a fast, versatile, and industrially established technique that can provide a highly uniform coating of particles on CN thin films supported by conductive substrates. Subjecting a suspension that contains partially charged particles (such as metals or metal-oxides and -hydroxides) to an applied electric field drives the cocatalyst particles to the electrode’s surface, resulting in a uniform coating at room temperature. For example, we used EPD to deposit ZnSe nanocrystals into a porous CN-based photoanode.^98^

#### 3.3.3 Solvothermal

This technique enables the direct modification of thin films under elevated-pressure conditions above the solvent’s boiling point, potentially resulting in intimate cocatalyst–photocatalyst contact. For example, we deposited a metal organic framework (Ni/Fe-MIL-53) onto a porous CN film. After electrochemical activation, it converted into an OER cocatalyst (nickel–iron-oxyhydroxide).^94^

#### 3.3.4 Single-Atom Photocatalyst (SAPC)

Teng et al. prepared antimony SAPC by a simple bottom-up method.^100,101^ This method can be easily adopted for current CN PCP preparation protocols, either by depositing presynthesized Sb-SAPC-containing CN powders or by *in situ* growth using an Sb-containing CN precursor.

## 4 Precursor/Substrate Interplay for CN Films and Panels

The structure and physicochemical properties of CN layers formed by deposition of CN powders are similar to the parent CN powders used for coating, unless the binder plays a significant role. However, the *in situ*-grown photoactive layers may exhibit compelling properties since the formation process occurs on a heated substrate.^102^ Therefore, in this section, we overview the observed properties of CN layers prepared by the *in situ* approach. For the properties of CN layers prepared by other *in situ* grown approaches, we refer the reader to the review by Bian et al.^103^

### 4.1 Surface Morphology

Surface morphology is conveniently analyzed using scanning electron microscopy (SEM). The nature of the CN precursor can direct the CN growth on the substrate’s surface. For example, in our recent work,^15^ we observed that a supramolecular precursor (MCA) yields CN with a rod morphology (CN_MCA_), whereas melamine results in the “common” CN morphology (CN_M_). After introducing an additional doctor-blade step during CN_MCA_ synthesis, the obtained CN_MCA‑D_ exhibited a regular porous morphology. Furthermore, cross-sectional SEM analysis helps estimate the average thickness of the CN layer. For example, CN_MCA_ (without a doctor-blade step) showed a dense layer of CN rods with an average thickness of approximately 1.6 μm. On the other hand, adding the doctor-blade step yields a dense, porous CN layer with variable thickness (ca. 30–70 μm in this work). Overall, such analysis demonstrates how the choice of CN precursor(s) and deposition method may affect both morphology and coating thickness.^15^ In the rapid-heating study,^16^ the 3MelM supramolecular precursor yields a round, cage-like morphology for CN-10 (∼20 μm thick), owing to procedure modification.

Besides the precursor choice, the type of substrate also dictates the CN’s surface morphology. For example, growing CN on a KCl underlayer resulted in a sponge-like CN structure, whereas using plain formed irregular, elongated structures using the same melamine precursor.^19^ Bian et al. observed that the CN surface morphology was substrate-dependent; FTO and ITO yielded continuous films with nanoparticles on their surfaces, whereas glass yielded CN rods.^68^

Further investigation into the interplay between the substrate’s chemical composition and roughness, CN precursor(s), and the experimental growth parameters is needed. Fast surface characterization methods, such as laser-scanning confocal microscopes and ellipsometry, will help establish these relationships and enable assessment of surface roughness and thickness, critical for large surfaces and reproducibility.

### 4.2 Structural Characterization

Fourier-transform infrared (FTIR) spectroscopy is a rapid analytical tool for qualitatively determining surface group functionalities. Of special interest are the features a CN layer exhibits upon modification of its precursor(s). For example, we showed that only supramolecular MCA precursors resulted in a CN with cyanide defects, whereas CN_M_ (prepared only from melamine) exhibited only “regular” CN features.^15^ Interestingly, the bottom layer of CN_MCA‑D_ (adding a doctor-blade step during CN_MCA_ synthesis) exhibited these cyanide defects, whereas the upper layer did not. On the other hand, bulk CN powder (MCA precursor calcination in a ceramic crucible, in the absence of a substrate) was free of cyanide defects. It indicates the combined role of a supramolecular precursor and glass substrate on the final CN structure.^15^ A different mechanism for cyanide defect introduction was demonstrated in our melamine vapor-phase deposition over KCl underlayers. In this case, potassium doping was responsible for the defects using simple melamine as the precursor.^19^

The (powder) X-ray diffraction (XRD) analysis is a standard tool to determine the type of carbon nitride one synthesizes. For polymeric CNs, two expected signals appear: the stronger at about 27.4° (attributed to the (002) plane) and the weaker ca. 13.0° (attributed to the (100) plane); these characteristic signals are common in g-C_3_N_4_ and melon polymorphs.^104^ In the case of CN_MCA_ panels, we did not observe noticeable diffraction signals.^15^ Though it might indicate a lower degree of repetitive stacking, possibly originating from the rod morphology, it is more likely that the relatively thin layer (∼1.6 μm) is not sufficient to produce a signal under the experimental conditions. On the other hand, the CN_MCA‑D_ panel, featuring a dense, porous CN morphology with an average thickness of 37 μm, exhibited these characteristic XRD signals. In another study, Bian et al. observed that CN thin films formed on FTO, ITO, and silica showed similar XRD patterns, whereas the film on glass showed a shift toward lower angles, indicating an increase in the interlayer distance.^71^

### 4.3 Optical Properties

The morphology and thickness of CN layers might leave noticeable traces in the measured optical properties. Diffuse reflectance ultraviolet–visible (DRUV–vis) spectroscopy is equivalent, for thick films, to absorption spectroscopy (*via* the Kubelka–Munk analysis), which also allows optical band gap estimation using a Tauc plot.^105^ As a representative example, a CN_MCA_ film shows higher optical activity than a thicker CN_MCA‑D_ panel, especially at longer wavelengths (>450 nm), attributed to its rod-like morphology, which may result in multiple reflections of incident light.^15^ Furthermore, the increasing CN thickness (from ∼1.6 to ca. 37 μm) resulted in a dramatic decrease in transmittance percent (%*T*) from ca. 32% to ca. 0.2% about λ = 450 nm.^15^

Importantly, such measurements are a critical quantitative tool to (1) determine shifts in the optical band gap (for example, by changing the degree of polymerization, introduction of heteroatoms, and so forth), (2) determine the absorbed photon flux for quantum efficiency calculations. For thin films, it is customary to measure the absorptance (100%–%*T*–%*R*), *i.e.*, the absorbed percent of photons as a function of wavelength by measuring the film’s transmittance and reflectance (%*R*). When a CN film is of the order of tens of μm, transmittance becomes negligible, and optical property determination relies on DRUV–vis practices.

Bian et al. observed that the CN film’s thickness can influence the photoluminescence (PL). They showed similar PL spectra of CN over FTO, ITO, and silica (thickness in the 55–72 nm range), while CN/glass (1.3 ± 0.2 μm) resulted in a PL behavior akin to that of a CN powder.^71^

## 5 Photocatalytic Applications

Figure 6 depicts a typical photocatalytic mechanism for a CN panel. Figure 6a illustrates the simplest case, where incident radiation photogenerated electrons and holes in a CN film; they migrate to the surface, where both reduction and oxidation half-reactions occur. In contrast, in a PEC device, each half-reaction occurs at a different electrode, and an additional voltage bias is applied. In the case of a thick, porous CN film (Figure 6b), the excess thickness blocks light from reaching the CN near the substrate, forming an effective dark zone that is detrimental to charge photogeneration. The porosity allows reactants to reach reactive sites deep within the porous film and for products to diffuse out to the reaction medium. Since the reaction occurs at the solid–liquid interface within the porous matrix, it bypasses charge-mobility limitations. As shown in Figure 6c, the presence of dedicated cocatalysts provides active sites for the corresponding reduction and oxidation half-reactions, hastens the consumption of photogenerated carriers, and suppresses competing recombination processes, thereby improving the overall photocatalytic performance.

**Figure 6:**
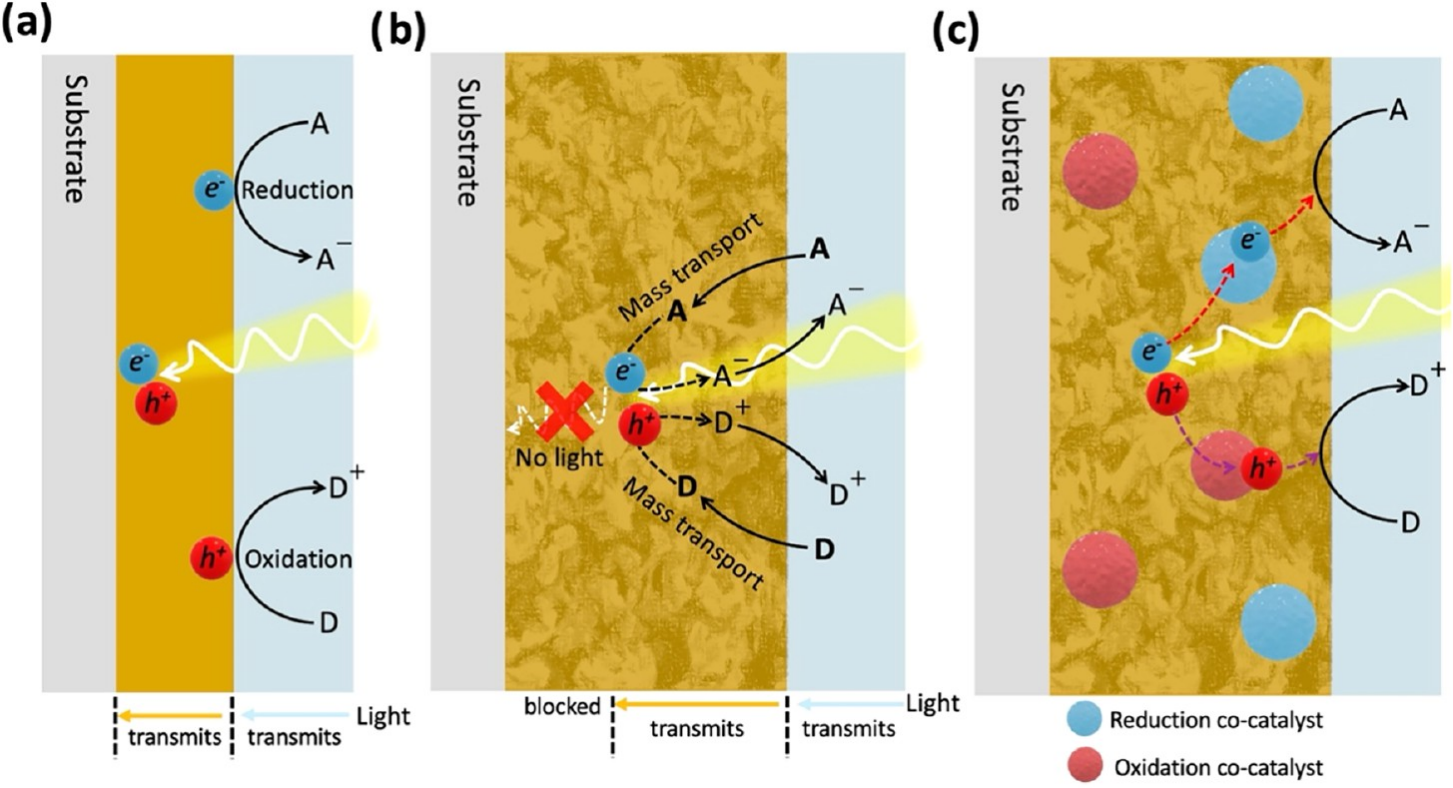
Photocatalytic mechanism over CN PCPs. (a) Surface photocatalytic reaction over a thin film, where the light transmits through the reaction medium (light blue) and into the CN to generate holes and electrons, which can subsequently oxidize a donor (D) molecule or reduce an acceptor (A) molecule, respectively. (b) In thick, porous CN layers, light transmission is effective (indicated by a yellow arrow) until it is attenuated, and the porosity allows mass transport of reactants and products during photocatalysis within the layer. (c) The presence of cocatalysts enhances effective charge separation and provides active sites for redox reactions.

As a semiconductor powder, CN can, in principle, perform both reduction and oxidation reactions; however, for reduction reactions, a cocatalyst such as Pt is usually required. Thanks to its suitable band positions and its ability to readily generate various reactive oxygen species (*e.g.*, superoxide radical (O_2_^·–^) and singlet oxygen (^1^O_2_)) from dissolved molecular oxygen (O_2_), CN has been explored as the photoactive layer in PCPs. These panels have already been demonstrated for several applications, including HER (in the presence of a sacrificial electron donor, also known as a hole scavenger), H_2_O_2_ production, 5-hydroxymethylfurfural (HMF) oxidation, C–H oxidation, and CO_2_ reduction, using either batch or flow reactor configurations.

### 5.1 Hydrogen Production (Reduction Half-Reaction)

In 2015, for the first time, Schröder et al. demonstrated large-scale H_2_ production under natural sunlight by immobilizing Pt@m-CN on SS plates configured in a flow reactor (Figure 2a).^3^ The Pt served as a cocatalyst while triethanolamine (TEOA) served as a hole scavenger. Within one month, around 18.2 L of H_2_(g) was produced, for which the PCP collected 83.8 kWh of energy from sunlight. The calculated rate for the entire period was 0.22 L kWh^–1^; a maximum solar-to-hydrogen (STH) conversion of 0.12% was achieved. This work strongly demonstrated the advantage and simplicity of CN-based PCPs for practical hydrogen production.

In this context, in 2021, Uekert et al. prepared scalable CN_*x*_|Ni_2_P panels,^14^ as discussed in Section 3.1.1. These CN_*x*_|Ni_2_P PCPs were first tested for maximal H_2_ yield, where a 1 cm^2^ panel was directly immersed in H_2_O containing EG and illuminated by a simulated solar light (AM 1.5G) at 25 °C under nitrogen. Here, the authors chose EG as a model substrate for their photoreforming (PR) experiments, as it is a monomer of poly­(ethylene terephthalate) (PET), which accounts for nearly 10% of global plastic waste. Ni_2_P was chosen as a cocatalyst. Using a front-irradiation configuration (light passes through the reaction solution), the CN_*x*_|Ni_2_P panel exhibited maximal H_2_ yield with a catalyst loading of 1.92 mg_CN*x*_ cm^–2^, resulting from better light management (Figure 7a) at optimal film thickness.

**Figure 7:**
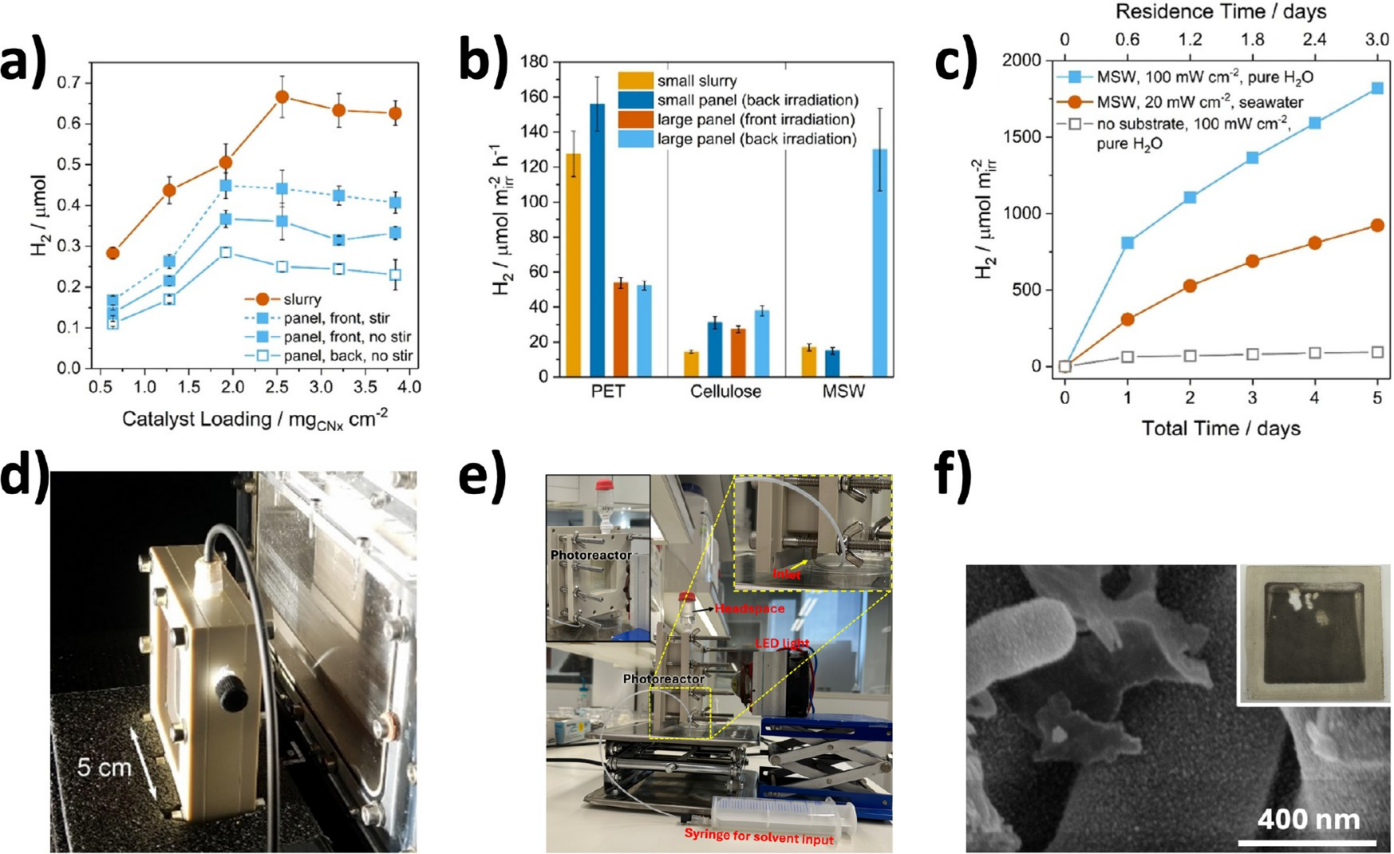
Reactor designs utilizing PCPs. (a) CN_*x*_|Ni_2_P loading optimization on PCPs for HER (EG PR). (b) Comparison of small-scale slurry, small-scale panels (1 cm^2^), and front- and back-irradiation configurations of large-scale panels (25 cm^2^) for PR of PET, α-cellulose, and MSW as the chemical substrates. (c) Scaled-up long-term HER. (d) The 25 cm^2^ photoreactor used in (a–c). (e) Photograph of a photoreactor setup used for HER in our group. (f) SEM image of a CN_MCA_ panel after HER in the photoreactor shown in (e); inset: postoperation photograph of CN_MCA_ PCP. (a–d) Reproduced with permission from ref 14. Copyright 2020 Wiley-VCH GmbH; (e–f) Adapted from ref 15. Copyright 2024 American Chemical Society.

The optimized CN_*x*_|Ni_2_P panel retained nearly 73% of the H_2_ yield in the slurry system and achieved an external quantum yield (EQY) of 0.021% ± 0.005% at λ = 430 nm. By introducing 600 rpm stirring, the optimized PCP achieved a H_2_ yield statistically equivalent to that of the slurry. This highlights lower efficiency due to the limited mass transport. On the other hand, under a back irradiation configuration (light passes through the frosted glass substrate), which is more practical for PR applications that deal with turbid solutions, the optimized panel showed only 20% lower H_2_ yield compared to a front irradiation configuration, because of light absorption by the frosted glass substrate.

The stabilities of the 1.92 mg_CN*x*_ cm^–2^ CN_*x*_|Ni_2_P panels in H_2_O or 0.5 M KOH­(aq) with EG were tested for 5 continuous days or in an intermittent 5-day regime, with recycling every 24 h. In H_2_O, the H_2_ areal efficiency decreased slowly from 110 to 81 μmol (H_2_) m^–2^ h^–1^ over 5 days and halved after the fourth reuse cycle. CN_*x*_ FTIR analysis showed an unchanged fingerprint region after operation, demonstrating chemical robustness; the authors attributed the decrease in efficiency to the degradation of the Ni_2_P cocatalyst, as they observed that 26% of the Ni content leaches into the PR solution after 5 days. Previously, the authors observed up to 60% of Ni leaching from a CN_*x*_|Ni_2_P slurry in H_2_O in the same time frame, suggesting the advantage of immobilizing CN_*x*_|Ni_2_P. In 0.5 M KOH­(aq), the CN_*x*_|Ni_2_P panels retained 70% activity after the fourth reuse cycle and exhibited only a small decrease in areal HER efficiency during continuous use. Under alkaline conditions, only 3% of the Ni content leaches into the PR solution after 5 days, due to the formation of a Ni­(OH)_2_ stabilizing layer over the Ni_2_P cocatalyst.

With these encouraging PCP results for PR of a model substrate (EG), the CN_*x*_|Ni_2_P panels (1.92 mg_CN*x*_ cm^–2^) were used for PR of various solid waste streams (PET powder, α-cellulose, and municipal solid waste (MSW)) as shown in Figure 7b. Under the back-irradiation configuration, 1 cm^2^ CN_*x*_|Ni_2_P panels produced 156 ± 15, 31 ± 3, and 15 ± 2 μmol m^–2^ h^–1^ of H_2_ during PR of PET, α-cellulose, and MSW, respectively, which are higher than, or similar to, slurries with the same amount of CN_*x*_|Ni_2_P. In the scale-up experiments, 25 cm^2^ CN_*x*_|Ni_2_P panels were used in a custom-designed reactor (Figure 7d), where 50 mL of pretreated waste was continuously circulated between the reservoir and the photoreactor at an optimum flow rate. The PR with PET showed similar H_2_ aerial efficiency in both front- and back-irradiation configurations due to the high transparency of the pretreated PET solution (80% transmittance at λ = 400 nm). However, a 3-fold drop in efficiency was observed upon scale-up from 1 to 25 cm^2^. On the other hand, the PR with α-cellulose showed a 40% higher H_2_ yield in the back-irradiation configuration than in the front-irradiation one, and exhibited similar H_2_ aerial efficiency at both scales. Lastly, the PR with MSW showed approximately 8–9 times improvement in efficiency upon scaling up from 1 to 25 cm^2^ (back-irradiation).

In the case of less transparent substrates in the reaction media—α-cellulose (16% transmittance at λ = 400 nm) and MSW (transmittance ca. 0 at λ = 400 nm)—the back-irradiation configuration is mandatory to achieve higher H_2_ efficiencies using 25 cm^2^ panels. This improvement, at least partially, stems from reactor design: In a small reactor, where a 1 cm^2^ PCP is placed against the reactor wall, the thin layer of waste solution between the photocatalytic film and the wall partially obstructs incident light. Upon scale-up, the photocatalyst is deposited on the 25 cm^2^ photoreactor window, which serves as the panel, thereby eliminating solution absorption (back-side illumination). In the case of transparent PET solutions, PR scaling may introduce additional factors, such as mass-transport challenges and irregularities in large PCPs, which can dominate and reduce H_2_ yield. Moreover, the choice of batch *vs* flow conditions may affect the adsorption and oxidation of reaction intermediates on the immobilized photocatalyst. Finally, to demonstrate the versatility and real-world applicability of the discussed PR system, the authors applied “worst-case” conditions (100% seawater, light intensity of 20 mW cm^–2^) to the scaled-up PR of MSW for 5 days and compared it to “ideal” conditions (100% pure water, simulated 1 sun, *i.e.*, 100 mW cm^–2^). As shown in Figure 7c, the 25 cm^2^ CN_*x*_|Ni_2_P panels produced half of the H_2_ production under “ideal” conditions. Overall, this work strongly emphasizes the importance of the following factors for photocatalytic process design and scale-up: photocatalyst loading (amount per area) or photocatalyst film thickness; reaction medium to enhance photocatalyst or cocatalyst stability; and reactor configuration, specifically the irradiation direction, to minimize unwanted light-absorption effects.

We have also demonstrated HER using a 6.7 × 6.7 cm^2^ CN_MCA_ panel (25 cm^2^ active irradiation area) combined with a Pt cocatalyst (*in situ* photodeposited) and EtOH as a hole scavenger in a closed-flow reactor (*i.e.*, batch conditions; shown in Figure 7e). After 4 h front-irradiation using a white LED, this PCP produced 5.84 μmol of H_2_ at a rate of 584 μmol g^–1^ h^–1^. Importantly, postoperational SEM analysis confirmed the CN’s stability and homogeneous distribution of the Pt cocatalyst (Figure 7f).

### 5.2 H_2_O_2_ Production (Reduction Half-Reaction)

Hydrogen peroxide (H_2_O_2_) has been gaining major attention as a promising carbon-free solar fuel. However, the existing anthraquinone process is energy-intensive and generates environmental pollution. Furthermore, this process also requires an external H_2_ feed. On the other hand, the photocatalytic molecular oxygen reduction reaction (ORR) to H_2_O_2_ offers a sustainable alternative process and does not require external H_2_. Along these lines, the previously discussed “sandwich” method CN PCPs,^15^ were tested for photocatalytic ORR (*i.e.*, H_2_O_2_ production) in a continuous-flow photoreactor (Figure 8a) from an aqueous solution (20% vol. EtOH) under an O_2_ atmosphere (Figure 8b). After 2 h front-illumination, the CN_MCA_ panel produced more H_2_O_2_ than CN_M_ and CN_MCA(1:1.5)_. Increasing the photocatalyst loading (specifically, the thicker CN_MCA‑D_) increased the ORR rates (H_2_O_2_ yield of 1023 μmol m^–2^), attributed to better light utilization. The estimated activity normalized to the amount of CN_MCA‑D_ is 117 μmol h^–1^ g^–1^. A similar reaction under batch conditions with CN_MCA‑D_ powder yields a comparable production rate of 91 μmol h^–1^ g^–1^. The H_2_O_2_ yield was further improved to 1628 μmol m^–2^ (242 μmol g^–1^ or 20 mM m^–2^) after 4 h using a CN_MCA‑D_ panel. Even though a precise comparison between the PCP’s activity in a flow configuration and state-of-the-art CN-based powder batch photocatalysis is not trivial, the CN_MCA‑D_ panel shows better ORR activity than many reported CN powders under related conditions.

**Figure 8:**
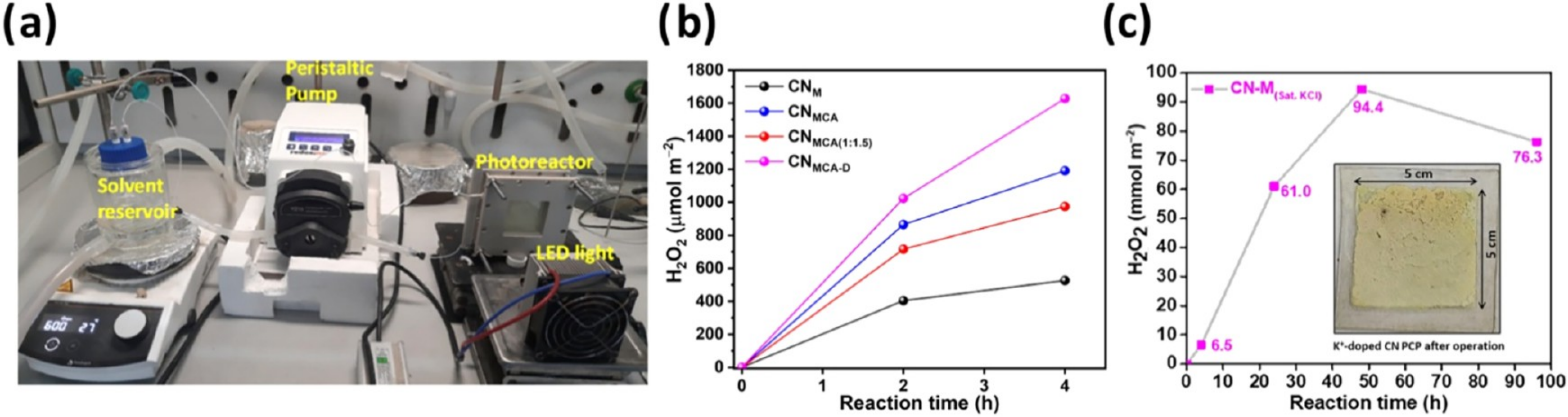
(a) Flow-reactor setup (25 cm^2^). (b) ORR using different CN panels using the system shown in (a). (c) Concentration of the photocatalytically produced H_2_O_2_ over K^+^-doped CN PCP; inset: image after 96 h of operation. (a–b) Adapted from ref 15. Copyright 2024 American Chemical Society; (c) adapted from ref 19. Available under a CC BY-NC-ND 4.0 license. Copyright 2026 The Author(s). *Small* published by Wiley-VCH GmbH.

The stability of the CN_MCA‑D_ panels was confirmed using FTIR and UV–vis spectroscopy. The SEM images obtained after 4 h of operation in a flow condition under illumination corroborate the physical stability of the CN_MCA‑D_ panels, indicating a strong interaction between the glass surface and the CN films.

In line with this work, our next study tested K^+^-doped CN PCPs (*via* vapor-phase synthesis) for H_2_O_2_ production.^19^ An optimized 2.64 cm^2^ K^+^-doped CN PCPs under batch conditions produced much higher H_2_O_2_ yield (1.8 ± 0.3 mmol m^–2^) compared to undoped CN PCP (0.2 mmol m^–2^) in 20% vol. EtOH aqueous solution after 4 h. In pure EtOH, the K^+^-doped CN PCP showed a significant improvement in the H_2_O_2_ yield (5.9 ± 0.7 mmol m^–2^), which is 18 times higher than the undoped counterpart. With these encouraging results in hand, we scaled up the PCP to 6.8 × 6.8 cm^2^ (Figure 5), scratched the edges to match the effective 25 cm^2^ illuminated area, and used it in the continuous-flow reactor (Figure 8a) for ORR in 100% EtOH for up to 96 h (Figure 8c). The H_2_O_2_ yield increased progressively up to a maximum around 48 h (94.4 mmol m^–2^). The panel remains physically stable (only minor detachment at the edges) even after 96 h of continuous operation under flow conditions. Detailed postcharacterization studies showed partial potassium leaching, underscoring the panel’s overall physical and chemical stability under these conditions.

We propose that future CN PCPs for H_2_O_2_ production should focus on eliminating the use of sacrificial electron donors entirely, a challenging feat given that several concurrent reactions must be balanced.^106^

Recently, Teng et al. used the Sb-SAPC, discussed in Section 3.3, toward H_2_O_2_ production from pure water, with an apparent quantum yield of 17.6% at 420 nm and a solar-to-chemical efficiency of 0.61% under batch conditions, using powder Sb-modified CN. They also showed long-term stability and scale-up concept using a fixed-bed reactor under natural sunlight.^100,101^

### 5.3 Hydroxymethylfurfural (HMF) Oxidation

The described continuous-flow photoreactor and the CN_MCA‑D_ panel enabled photocatalytic HMF oxidation in pure water. After 24 h (residence time of 15.6 h), the PCP converted nearly 75% of the HMF, yielding 13% 2,5-diformylfuran (DFF). The observed moderate DFF yield was attributed to the coproduction of H_2_O_2_, which presumably reacts with DFF—leading to the formation of CO_2_ or other aliphatic intermediates (including HCOOH and maleic acid). We are currently exploring methods to collect the H_2_O_2_ generated during HMF oxidation to improve DFF yield. However, the reported DFF yield is still comparable to that of conventional batch systems that use water as a green solvent. Interestingly, under continuous-flow conditions, we achieved a high HMF conversion rate (1185 μmol h^–1^ g^–1^), comparable to state-of-the-art heterogeneous photocatalysts.^15^

### 5.4 C–H Oxidation

Recently, our group (Abed et al.)^17^ prepared sulfur-doped CN (CN-CMBT_1_) PCPs to demonstrate toluene photooxidation, following encouraging results using analogous powders for photocatalytic toluene oxidation to benzaldehyde and benzoic acid. The experiments were initially performed in a batch reactor using a front-irradiation configuration, where 1.2 × 1.2 cm^2^ glass/CN-CMBT_1_ PCP was immersed in pure toluene in a reaction tube. After 11 days of illumination, it produced 7.48 μmol of benzaldehyde, 0.9 μmol of benzyl alcohol, and a trace amount of benzoic acid (0.001 μmol) (Figure 9a). Analysis using XRD and FTIR and UV–vis spectroscopies, after 11 days of operation, confirmed that the CN PCPs remain almost unaltered.

**Figure 9:**
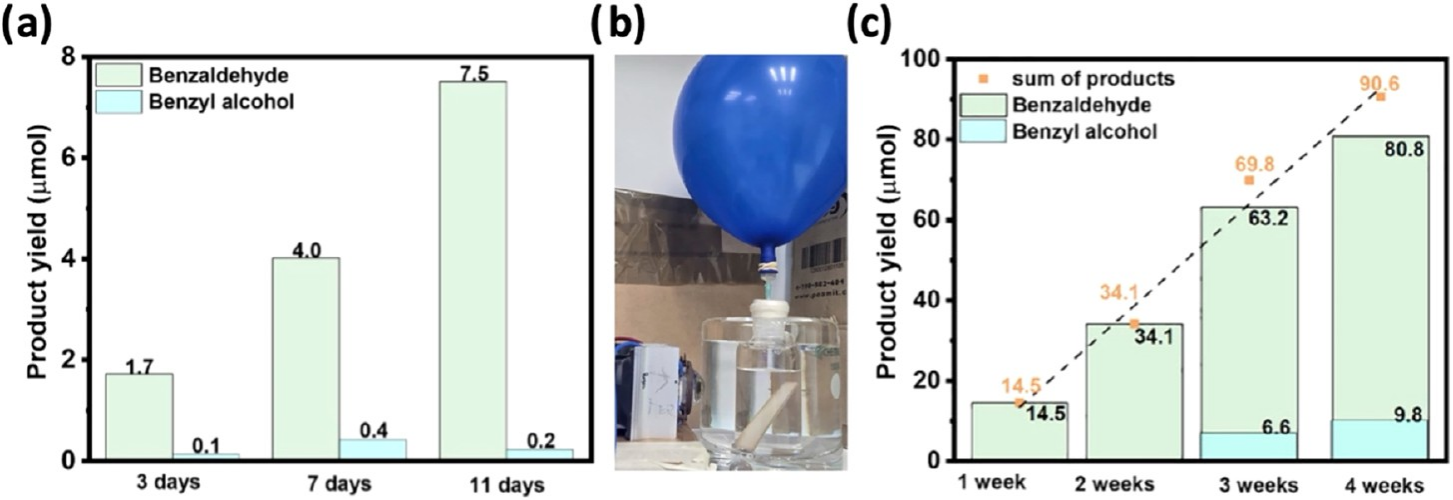
Toluene photooxidation using glass/CN-CMBT_1_ PCPs in pure toluene batch configuration. (a) using 1.2 × 1.2 cm^2^ PCP. 120 mL pure toluene batch reactor setup (7.2 × 2.0 cm^2^ PCP) used for long-term photooxidation: (b) Photograph, and (c) Product analysis. Adapted from ref 17. Available under a CC BY-NC-ND 4.0 license. Copyright 2025 The Author(s). *Small* published by Wiley-VCH GmbH.

A scale-up was demonstrated in a batch reactor (front-illumination) with a 7.2 × 2 cm^2^ PCP and 120 mL of pure toluene (Figure 9b). After 4 weeks, 80.8 μmol benzaldehyde and 9.8 μmol benzyl alcohol were produced without any traces of benzoic acid, and an ∼88.5 μmol m^–2^ h^–1^ average product formation rate (Figure 9c). Interestingly, these PCPs yielded benzyl alcohol as a minor product, which was not observed with CN-CMBT_1_ powder. We attributed this to a possible side reaction between two benzyl hydroperoxide intermediates, leading to the formation of benzyl alcohol and benzaldehyde over the panel.

Our group (Tashakory et al.)^16^ used CN-10 PCPs for cyclohexane (CHA) photooxidation and a commercial multiphotoreactor (390 nm LED; front-illumination; Figure 10a) for temperature-screening experiments, which revealed an optimum at 35 °C. We demonstrated in a biphasic system (Figure 10b) that hydrohalogenic acids improve yields and drive product selectivity (HCl favors ketone; HBr favors alcohol). This divergence was ascribed to a synergy of high acidity and halide identity. Notably, static PCPs offered an advantage over unstirred powders by ensuring continuous dual-phase contact, though slightly lower than that of continuously stirred CN powder (Figure 10c).

**Figure 10:**
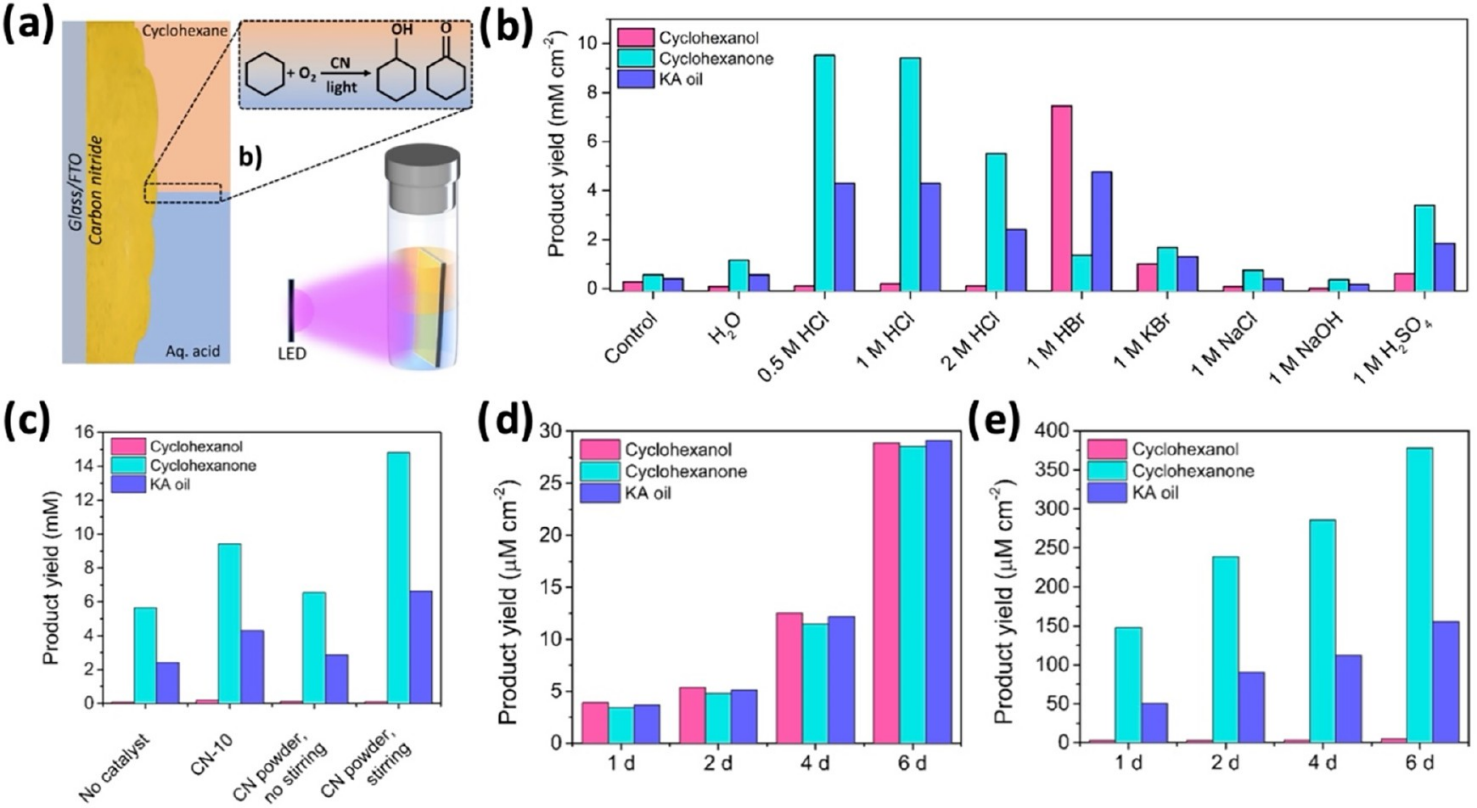
Cyclohexane oxidation using CN PCPs. (a) An illustration of a biphasic CHA–water system and a batch reaction setup. (b) Photocatalytic activity in a biphasic system. (c) Photocatalytic performance comparison of PCPs and CN powder—with and without stirring. (d) Scaled-up photocatalytic CHA oxidation over 12 cm^2^ PCPs for 6 days in pure CHA (45 mL), and in (e) biphasic CHA (35 mL) + 1 M HCl (10 mL). Adapted from ref 16. Available under a CC BY 4.0 license. Copyright 2025 The Author(s). *Photocatalysis* published by Scilight.

Finally, scale-up experiments were performed in a batch reactor with a 6 × 2 cm^2^ PCP in pure CHA (Figure 10d) and in biphasic CHA–HCl­(aq) (Figure 10e) for 6 days. In pure CHA, the yields of cyclohexanol and cyclohexanone were comparable; in the biphasic system, cyclohexanone was the major product. Overall, after 6 days in the biphasic system, the PCP produced around 5 μM cm^–2^ cyclohexanol, 378 μM cm^–2^ cyclohexanone, and 155 μM cm^–2^ ketone–alcohol (KA) oil.

The lower region (in contact with 1 M HCl) and the upper region (in contact with CHA) of the PCP were thoroughly characterized after operation, with no significant observed changes, except that delamination was evident in the upper region (in contact with CHA) in cross-sectional SEM imaging. It was ascribed to the interaction of CN with chlorine species, and nearly 1% at. Cl was detected at the CN’s surface *via* X-ray photoelectron spectroscopy (XPS).

### 5.5 CO_2_ Reduction

After obtaining encouraging results with CN_*x*_–ITO|FDH photocatalyst powders for combined CO_2_ reduction to formate and 4-methoxybenzylalcohol oxidation to the corresponding aldehyde (MBAld), Rahaman et al. prepared various CN_*x*_–ITO|FDH photocatalyst sheets for combined CO_2_ reduction and alcohol oxidation.^18^ Among the tested sheets (different CN_*x*_/ITO ratios), the 1:3 CN_*x*_/ITO ratio showed the best performance and produced 21 ± 3 μmol_formate_ cm^–2^ and 18 ± 0.1 μmol_MBAld_ cm^–2^ after 10 h solar-simulated irradiation. After this composition optimization, 200 pmol of FDH loading was found to be optimal. Next, the authors tested this optimized CN_*x*_–ITO|FDH biohybrid on different glass substrates (ITO- or FTO-coated and frosted glass). Similar activity was observed for ITO and FTO, whereas frosted glass showed lower activity due to the mechanical instability of the films. Later, the authors investigated the role of conductive ITO particles by replacing them with MO particles—insulator (ZrO_2_ and SiO_2_) or semiconductor (TiO_2_), which showed low activity. This served as a corroboration of the ITO’s role in improving solid-state electron conductivity.

The recyclability experiments showed a decline in activity during the third–4th cycles. Furthermore, the activity was not recovered in the fifth cycle, even after ozone treatment. They attributed the diminishing performance to morphological changes observed after operation, as determined by scanning/transmission electron microscopy (S/TEM) with energy-dispersive X-ray spectroscopy (EDS).

In a scale-up experiment, the authors submerged a 50 cm^2^-optimized sheet in a CO_2_-saturated solution within an airtight, transparent Perspex photoreactor. The photocatalytic experiment was conducted on a rooftop for 3 days under natural sunlight. Their time-dependent product analysis revealed that the formate and MBAld yields increased gradually in a ∼ 1:1 ratio.

## 6 Knowledge Transfer from CN Powders and PEC Films to Photocatalytic Panels

Since CN films acting as the PCPs’ photoactive layers offer operational and performance advantages over CN powders, to further develop this relatively new architecture for carbon nitride-based materials, we envision that the chemistry of *in situ*-grown CN panels is going to advance by adapting the vast existing knowledge from CN powders or CN photoelectrodes, specifically tailoring the intrinsic layer’s properties, design of different heterostructures, including cocatalysts, and more as illustrated in Figure 11 and elaborated in the following subsections.

**Figure 11:**
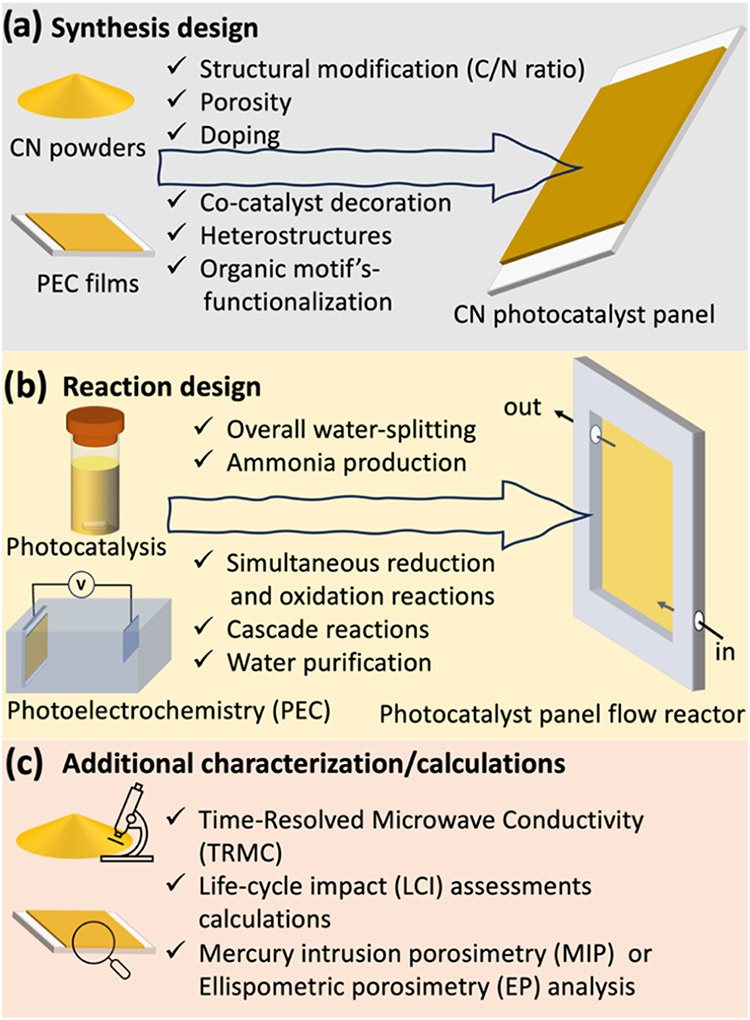
Prominent knowledge transfer opportunities from CN powders or from PEC photoelectrodes to CN-based PCPs. (a) Synthesis design, (b) Reaction design, and (c) Additional characterization/calculations.

### 6.1 Synthesis Design

CN materials generally suffer from low conductivity, rapid charge recombination, low specific surface area (*S*_A_), limited visible-light response (due to a band gap of approximately 2.7 eV), and a lack of specific reaction sites. Extensive research was conducted to overcome these limitations, and the various design strategies should be easily transferable.

#### 6.1.1 Structural Modification

The structural composition of g-C_3_N_4_ (*i.e.*, an atomic C/N ratio ca. 0.75) can be tuned by using different CN precursors. Different single and multiple precursors have been used to produce CN with low to high N content (C_*x*_N_*y*_), where *x*/*y* ≠ 0.75. Reviews by Kumar et al.^107^ and Vidyasagar et al.^108^ highlight the advantages of these C_*x*_N_*y*_ materials for energy- and environment-related applications. Thus, various CN precursors can be used during *in situ* deposition to produce photocatalyst panels with different stoichiometries and N moieties (−NH_*x*_ and −CN). Moreover, numerous established methodologies exist for incorporating comonomers and carbon-rich materials during thermal polymerization to enhance carbon content.^9,109,110^

#### 6.1.2 Porosity

The *S*_A_ of CNs deposited on substrates can also be improved to enhance photocatalytic activity. However, unlike powder synthesis, the so-called templating route (hard and soft) to induce porosity during the *in situ* deposition of CN on substrates is challenging, since the template or its removal can affect the substrate–CN interaction. However, single-step calcination of CN supramolecular precursors^17^ or pretreated CN precursors (ammeline and ammelide)^111^ can produce porous CN powders without the aid of any additional template. Thus, these precursors can be further optimized, and new precursors can be explored during CN panel synthesis.

#### 6.1.3 Doping

Doped CNs (with metal and nonmetal elements) have gained significant interest for various photocatalytic or PEC applications. Generally, doping alters the photophysical properties and unveils improved catalytic activity compared to bare CN. Along these lines, we explored several dopants and doping strategies for CN synthesis: Barrio et al. utilized crystal design to embed dopants—such as Na, K, or both—into the ensuing CN;^112^ Shmila et al. developed Na- and B-doped CN photoanode (CN_TUB_) by direct growth of CN monomer from a hot precursor solution;^92^ Garg et al. developed KCl-doped CN photoanodes using solvothermal conditions (CN-CMK-*s*-MeOH);^96^ Abed et al. prepared S-doped CN photocatalyst powder and panels.^17^ Other groups also explored various dopants; for example, Zhou et al. reported a P-doped CN photocatalyst,^113^ and Shah and Gilbertson reported an O-doped CN.^114^ Moreover, to lower the environmental impact, biochar-tailored strategies can be implemented in CN PCP synthesis to induce C-bridging and N-defects to the CN structure.^115^ Overall, the introduction of these dopants resulted in higher performance metrics than their pristine counterparts, demonstrating the advantage of adopting these strategies for future CN panel synthesis.

#### 6.1.4 Co-Catalyst Decoration

As discussed in Section 5 and Figure 6, attaching cocatalysts to improve charge separation and hinder prominent charge recombination in CNs is viable. A suitable cocatalyst provides active reaction sites for reduction (*e.g.*, HER, CO_2_ reduction, N_2_ reduction) or oxidation (*e.g.*, OER). In a recent review, Li et al. classified various cocatalysts—metals, metal compounds, nonmetals, metal–metal compound hybrids, and metal–carbon hybrids—used in photocatalysis and summarized the charge-transfer mechanisms at the semiconductor/cocatalyst interface based on energy-band theory.^116^ Metal single-atom cocatalysts, due to a lack of inactive internal metal atoms, showed exciting improvements in photocatalytic HER when compared to the “regular” metal cococatalysts.^117^ In another study, a high-loading Ni single-atom photocatalyst showed efficient H_2_O_2_ synthesis in water.^118^ Furthermore, noble-metal-free plasmonic systems based on titanium-nitride (TiN) nanoparticles showed efficient solar CO_2_ reduction.^119^ Our group added Y clusters into the CN acting as an oxidation cocatalyst.^97^

Llovet and co-workers recently explored strategies to attach or chemically anchor Ru-based molecular OER cocatalysts to CN photoanodes, achieving higher PEC performance.^120,121^ Iron-based molecular cocatalysts for CN were also shown to improve CO_2_ reduction.^122^ Thus, future CN panel synthesis could benefit from adapting such protocols to form tailored CN/cocatalyst interfaces, thereby achieving higher activity and stability in PCPs for the sought reactions. For example, the decoration of noble-metal-free single-atom cocatalysts or plasmonic nanoparticles, as well as molecular cocatalysts on CN, may prove an exciting pursuit.

#### 6.1.5 Heterostructures

It is impossible for a single pristine CN to harvest the full spectrum of sunlight, support charge separation, and exhibit efficient redox activity.^123^ Thus, constructing CN homojunctions (*i.e.*, two types of CN, such as doped and undoped) and heterostructures between CN and another suitable photocatalyst can address these difficulties.^123^ Figure 12 illustrates CN/MO-based heterojunctions (type-II or Z-scheme) and associated charge migration. Such a configuration may allow killing two birds with one stone: improving light harvesting and reducing recombination by spatially separating photoexcited charge carriers. Importantly, these architectures may prove pivotal for achieving the challenging overall water-splitting or CO_2_ reduction, most probably, in conjunction with a suitable cocatalyst(s). The scheme is not limited to MOs, as the second semiconductor may be another type of CN (sometimes referred to as a homojunction).

**Figure 12:**
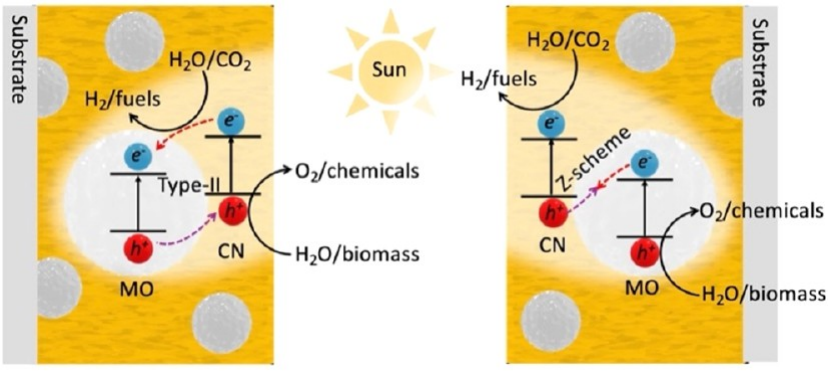
CN/MO type-II (staggered energy levels) or Z-scheme heterojunction PCPs.

In some cases, thermal polycondensation of CN supramolecular complexes can yield amorphous triazine–heptazine-based CN allotropic heterojunctions.^124^ We developed CN-based photoanodes with homojunctions and heterojunctions for PEC applications and observed significant performance improvements.^5,67,85,92,98^ In separate works, we observed that blending reduced graphene oxide (rGO) into various CN-based photoanodes greatly improved the stability of the CN electrode and the photocurrent density.^93–95^ The presence of rGO improved the electron diffusion and conductivity.^125^

Thus, CN-based junctions and blended conductive nanomaterials such as rGO are a viable route to improve light harvesting and charge separation in PCPs. Notably, unlike photoelectrodes, in the case of PCPs, there is no conductive back-contact that accepts charge carriers, which makes layered heterostructures less appealing and motivates further exploration of blended junctions with other CNs, semiconductors, conductive carbons, and so forth.

#### 6.1.6 Organic Motifs’ Functionalization

Modification of CN’s molecular structure by covalent functionalization of an organic molecular motif was extensively pursued to improve CN’s photocatalytic activity.^126^ Recently, Peng et al. modified the surface of a CN nanolayer with a benzylamino group.^127^ This modification improves visible-light response, charge separation, and O_2_ adsorption, demonstrating an impressive H_2_O_2_ yield from pure water in a flow system. Thus, covalent functionalization strategies can be adapted for use on CN PCPs. Furthermore, exciting photocatalytic strategies to graft cyano groups onto CN to yield cyanidated CN can be adapted to CN PCPs.^128^

#### 6.1.7 Other

The salt melt synthesis (SMS) is an excellent method for obtaining highly crystalline alkali-metal cation-intercalated carbon nitrides as the poly­(triazine imide) (PTI) type. Liu et al. used a binary salt mixture (LiCl and NaCl) to produce highly crystalline PTI-LiNa and achieved overall water-splitting.^129^ We expect that SMS adaptation for PCPs could achieve similar merits.

Merging CN polymorphs with other polymers is another type of organic heterojunction, or a CN/polymer hybrid material (for example, CN embedded in hydrogels or CN functionalized with polymers or modified with donor–acceptor pairs). Cao et al. reviewed different CN/polymer fabrication methods and their applications.^130^ This strategy can yield improvements in PCPs, analogous to the ones discussed in the heterojunctions section. Moreover, it can enable advanced photoreactor design, as polymers can act as porous supports that can be integrated into devices as membranes, floating self-standing catalysts, foams, and more; see, for example, the configurations in Figure 1b–d,i,j, and l.

A significant limitation of the various *in situ* film growth methods is the requirement for thermal condensation/polymerization, which typically occurs above 500 °C. Modifications to the sought-after CN polymer,^131^ or heating method^86^ may enable lower synthetic temperatures, which, if successful, could facilitate deposition on flexible plastic substrates and potentially lead to novel photoreactor designs.

### 6.2 Reaction Design

As discussed in earlier sections, CN PCPs have thus far been used for only a limited number of photocatalytic applications compared to CN powders. Therefore, there is ample room to expand the scope of reactions to which CN PCPs can be applied, with reports on powder photocatalysts demonstrating their feasibility. However, most photocatalytic reactions focus on one-half-reaction (either reduction or oxidation), and the other half-reaction is often replaced by sacrificial reagent consumption. The use of these sacrificial reagents adds extra cost to the system, requires a continuous supply, and generates unwanted side products (often mixed with the desired product or compete with the counter half-reaction). Thus, using these sacrificial reagents should be replaced with cost-effective reagents or waste substrates. For example, Reisner’s group demonstrated the use of substrates derived from biomass and plastic to H_2_ ^132^ and H_2_ along with HCOO^–^.^133^ Thus, CN PCPs should focus on using both half-reactions effectively to generate solar fuels or chemicals. Such reactions include, but are not limited to, overall water-splitting,^134^ ammonia production,^135^ simultaneous reduction and oxidation reactions,^136^ and water purification.^137^

Recently, we demonstrated a cascade reaction in which a CN photocatalyst produces H_2_O_2_ (*via* ORR of O_2_ dissolved in the reaction medium) and HCHO (the product of methanol oxidation), which subsequently react with each other to form H_2_ and HCOOH.^138^ This cascade approach enables even an HER-inactive photocatalyst (WO_3_) to produce H_2_. In another work, Meng et al. produced cyclohexanone oxime from cyclohexanol and NO_*x*_^–^, where cyclohexanol oxidizes to cyclohexanone and NO_*x*_^–^ reduces to NH_2_OH, which then combine to give cyclohexanone oxime.^139^ We propose to design CN PCPs to perform such cascade reactions. Furthermore, due to the advantage of flow systems, tailored CN-based panels can be applied for multistep organic transformations. Interestingly, the use of CN panels can also be extended to bacterial disinfection technologies.^140^

In terms of reactor design, compound parabolic concentrator-type systems can also be adapted to maximize sunlight utilization and achieve competitive product yields.^141^ Adapting piezoelectric photocatalytic systems is another strategy to induce charge separation, ultimately leading to high product yields.^115^

### 6.3 Additional Characterization/Calculations

Time-resolved microwave conductivity (TRMS) studies can be applied to CN PCPs to analyze the properties of photoinduced charge-carrier dynamics. This technique is advantageous for photocatalytic systems because it does not require a conductive substrate, making it uniquely suitable for PCP characterization, and can clarify whether and which photogenerated charge carriers contribute to photocatalytic activity. Recently, we employed this technique to investigate the effect of K^+^-doping on the photocatalytic activity of CN PCPs. We established a correlation between the optical absorptance and the TRMC signal, proving that the additional absorbance below the band edge of pristine CN is indeed translated into photogenerated charge carriers.^19^ This technique can complement advanced photophysical characterization, such as time-resolved transient absorption measurements, which are difficult for CN systems due to the characteristic short lifetimes.^90,97,98,121^

The *S*_A_ and porosity analysis of CN layers can be correlated to their photocatalytic activity. Standard N_2_ physisorption isotherm measurements at 77 K, widely used for CN powders, can be performed by placing scratched CN powder in standard measuring tubes (under the assumption of sufficiently high *S*_A_, to yield an absolute surface area of ca. 1 m^2^, to allow credible measurement). In other cases, mercury intrusion porosimetry (MIP) can be used to directly assess the porosity and pore-size distribution of porous CN films, as has been explored for photocatalytic concrete materials.^142^ The MIP complements “standard” physisorption by characterizing larger pores, which become significant when mass-transport phenomena are to be explored. Nondestructive ellipsometric porosimetry (EP) can be adapted for extremely thin, optically transparent CN films.^143^ Chemisorption measurements are another related technique that is insightful for specific reactions, such as CO_2_ reduction.

Life cycle impact (LCI) assessment calculations can provide a comprehensive environmental impact assessment of the PCP’s cradle-to-grave lifecycle, encompassing synthesis (manufacturing), operation, and, in some cases, disposal. We recently performed LCI calculations for CN and CN/Pt powder photocatalysts.^138^ In this system, the electricity used for synthesis was the primary contributor to the environmental impact. Nitrogen gas and precursors (melamine and cyanuric acid) contribute significantly to the ozone-depletion impact category. The H_2_PtCl_6_ (Pt cocatalyst precursor) makes a significant contribution to several impact categories. Thus, such LCI calculations can be crucial for designing CN PCPs, enabling the selection of sustainable materials and methods to minimize negative environmental impact.

## 7 Conclusions

Despite variations in preparation methods, CN panel syntheses share a common goal of achieving robust (thin) film coatings with efficient light-harvesting ability. The panels successfully demonstrate various photocatalytic applications, including hydrogen production (HER), hydrogen peroxide production (*via* ORR), biomass oxidation, C–H activation (toluene and cyclohexane oxidation), and CO_2_ reduction. The different panels consist of metal-free CN-only designs and hybrid films, paving the way for further improvements in activity and stability. PCPs can operate in organic (polar and nonpolar) and aqueous solutions, with varying pH values, and even in biphasic systems. The cost and environmental impact associated with the preparation of these panels can be offset by their recyclability and by increasing their size for more efficient photoreactor designs.

While the versatility of such panels, especially for photooxidation reactions, has been studied mostly with the available batch or flow reactor designs on a lab scale, further advancements in terms of photocatalytic efficiency, recyclability, and scalability are highly desired, which could trigger a strong momentum toward practical continuous-flow reactors equipped with these panels for fine-chemical synthesis. If such systems maintain competitive product yields for several years compared to a prototype reactor equipped with at least 1 m^2^ of CN-based panel, then they could be considered for commercial installation.^144^

## 8 Challenges and Perspectives

The deposition of synthesized CN powders offers versatility, enabling the deposition of any CN powder and building on the vast body of knowledge developed over nearly two decades of CN research. However, from a sustainability perspective, avoiding the use of a binder should improve the panel’s stability and performance, offsetting the costs and environmental impact associated with panel preparation.

For research purposes, the ease of preparation can be leveraged to screen various CN materials and hybrid structures, while paying special attention to the application of casting techniques to avoid irregularities. We believe that scaling up these methods would be counterproductive in most cases; after an initial success, efforts to develop binder-free deposition or *in situ* growth of the discovered CN compositions or configurations would prove fruitful. We identify the following topics as crucial for further development of CN PCPs:

### 8.1 Synthesis and Design of CN PCPs

For *in situ*-grown CN panels on substrates, understanding the interactions between CN precursors or their condensed intermediates and the substrate’s surface during growth is an important research avenue, despite the difficulties encountered in investigating material growth mechanisms. A better understanding of the substrate–CN interaction would improve adhesion and enable better control over the properties of the resulting CN.

The straightforward research path is to build upon the existing knowledge of pristine CN PCPs and implement the advanced structures discussed in Section 6.1 (such as CN modification, heterostructures, and cocatalysts), focusing on blends of thick, porous layers to improve light absorption and utilization, conductivity, charge separation, intermediate adsorption, and other properties. For challenging photocatalytic applications where two photocatalysts (CN and MO, as a representative example) are needed, the *in situ* growth CN methods can be combined with the EPD technique to deposit MO on the formed CN panel by any of the described *in situ* growth CN methods, resulting in a CN–MO heterostructure system (type II or Z-scheme).

### 8.2 Scale-Up and Industrial Applicability

To become applicable to industry, CN PCPs should meet several criteria: (a) Cost-effective synthesis, (b) efficient light harvesting, (c) long-term operational stability and activity, and (d) proven recyclability.

#### 8.2.1 Cost-Effective Synthesis

Since large areas of CN PCPs are to be fabricated, the development will include more energy-efficient manufacturing to reduce costs. For pilot-scale and larger systems, direct CN growth is more likely to be implemented than CN powder deposition (as detailed in Section 3.2), with the aim of aligning precursor deposition with commercial fabrication *via* proven industrial methods such as EPD or automated roll-to-roll processing.

#### 8.2.2 Efficient Light Harvesting

This is the primary requirement to improve achievable reaction rates (as detailed in Section 5).

#### 8.2.3 Long-Term Operational Stability

Established studies on the long-term operation of CN PCPs are still missing. We believe these studies will become available as the field grows. For industry applicability, this aspect should be prioritized, including detailed *post-mortem* analysis of the panels to assess the readiness of CN PCPs integration into pilot-scale photoreactors, determining the expected real-world performance metrics (temporal, long-term, activity) under operational conditions (generated heat, UV radiation, environmental factors), leaching of photocatalyst, cocatalysts, and other ingredients to asses safety, potential reuse, and recyclability.

#### 8.2.4 Recycling

Since the CN changes after prolonged photocatalysis, recycling strategies are to be determined. We propose that used CN can be converted into activated carbon, which has widespread applications, or break them down into possible CN monomers to produce ‘recycled’ CN or related materials. Interestingly, CN has already been used for CO_2_ capture and conversion to cyclic carbonates.^145^

### 8.3 Photocatalytic Equipment Compatibility and Design

Since the photocatalytic reactor design is nonstandardized, each research group designs its own reactor. For batch configurations (Figure 1a–g), the design is straightforward, with the main issues being uniform, known illumination; maintaining a stable temperature and reaction-oriented atmosphere (*e.g.*, air, O_2_-free); and ensuring accurate sampling. However, moving to continuous-flow designs (Figure 1i–k) introduces additional challenges. The first is how to sample the reaction without requiring a pause and without harming the atmosphere. The second challenge arises when nonaqueous or highly corrosive solutions are involved. For example, dedicated construction materials and tubing are required for organic media. In addition to stability, the leaching of organics from plastic components complicates the analytical characterization of reaction intermediates and product analysis of products.

Moreover, for efficient flow photoreactor design, many topics from the chemical engineering realm must be considered, such as flow regime, pressure drop, mixing efficiency, mass transport in boundary layers, residence time, and so on. These factors are free parameters that are mostly unexplored in CN-based systems. Finding the optimal operational temperature and maintaining it are additional challenges, especially in flow systems. If CN PCPs prove stable, operating at elevated temperatures should improve reaction rates and be viable, even at the expense of shorter-term operation, and might find use cases through thermal coupling with heat-emitting industrial effluents, especially for environmental remediation processes.

### 8.4 Photocatalytic Performance Metrics

For a reasonable comparison of photocatalytic activity between groups, we suggest including metrics such as solar-to-chemical conversion (SCC), quantum yield (QY), and wavelength-dependent action spectra. It is not trivial, as it requires dedicated equipment to measure the irradiance of the illumination source and the intensity that reaches the PCP (external QY). When the absorbed photon flux can be calculated (knowing the sample’s wavelength-dependent absorbance), internal QY can also be calculated. Moreover, the various types of possible illumination sources (Hg and Xe lamps (optionally coupled to AM 1.5G, bandpass, long-pass, or other filters), “white” and specific-wavelength LEDs) and different power ratings make it challenging to compare reported activities. Therefore, it is essential to know the illumination source, filters—if used, experimental configuration, operational conditions, and the source spectrum whenever possible. To establish comparable and meaningful figures of merit, we encourage reporting, for each reaction type and photocatalytic architecture, both an initial reaction rate and a reaction rate after prolonged operation, both normalized to the effective PCP geometric area, along the wavelength-dependent incident photon flux and its conversion efficiency. Postoperational analysis (“*post-mortem*”) of the used PCPs should be conducted in detail to uncover the degradation mechanisms. The adhesion of CN films and leaching, especially during organic transformations, are critical. For lab-scale experiments, *post-mortem* analysis after long-term operation would help uncover the degradation mechanisms, especially as the field matures and stable operation of weeks to months becomes common.

### 8.5 Performance of CN PCPs *vs* Powder Suspensions

PCPs offer better light management, easier product separation, and improved catalyst recovery. However, in terms of photocatalytic performance, they still need to compete with slurry-based systems. Slurry systems provide better mass transport and active *S*_A_, resulting in higher reaction rates. From a scale-up viewpoint, PCPs show some advantages over slurry systems. Thus, we propose that PCPs can be optimized to achieve high reaction rates comparable to those of slurry systems by inducing porosity to improve mass transport and by optimizing photoreactor design to achieve activity normalized to irradiance or effective area. Another research path is using higher-intensity light sources (solar concentrators), which can greatly enhance PCP photocatalytic activity.

### 8.6 Promising Photocatalytic Architectures

Among the reaction schemes mentioned in Section 6.2, we identify three significant methodologies that could lead to a leap forward in reaction scope and the long-term practical operation of CN-based PCPs.

(1) Since CN films are immobilized and do not require external stirring in a batch reactor, biphasic solvent systems are promising for organic transformations, thereby improving selectivity and product separation.^16,33^

(2) Cascade reactions can be utilized, where two distinct photocatalytic products form over the PCP and react with each other to produce a different product(s), as we have recently demonstrated for powder CN photocatalysis of hydrogen and formic acid from methanol oxidation and ORR.^138^
(3) By connecting in series flow-reactors, equipped with various PCPs, multistep organic transformations can be performed. For example, a product from one reactor (A) can serve as the feed to another reactor, yielding a distinct final product (B) in a single device with high conversion. This approach avoids the additional collection and feeding of A that is often required in two individual batch reactions.

In future scenarios, PCP-based continuous-flow reactors can be integrated with electrolyzers, enabling both systems to complement each other and achieve complex organic transformations, such as C–N coupling reactions through combined CO_2_ and N_2_ reduction. Furthermore, these configurations could be used for water treatment *via* Fenton-type chemistry.

## References

[1] Lang X., Chen X., Zhao J. (2014). Heterogeneous Visible Light Photocatalysis
for Selective Organic Transformations. Chem.
Soc. Rev..

[2] Xiao J., Liu X., Pan L., Shi C., Zhang X., Zou J.-J. (2020). Heterogeneous
Photocatalytic Organic Transformation Reactions Using Conjugated Polymers-Based
Materials. ACS Catal..

[3] Schröder M., Kailasam K., Borgmeyer J., Neumann M., Thomas A., Schomäcker R., Schwarze M. (2015). Hydrogen Evolution Reaction in a
Large-Scale Reactor Using a Carbon Nitride Photocatalyst under Natural
Sunlight Irradiation. Energy Technol..

[4] Nishiyama H., Yamada T., Nakabayashi M., Maehara Y., Yamaguchi M., Kuromiya Y., Nagatsuma Y., Tokudome H., Akiyama S., Watanabe T., Narushima R., Okunaka S., Shibata N., Takata T., Hisatomi T., Domen K. (2021). Photocatalytic Solar
Hydrogen Production from Water on a 100-M^2^ Scale. Nature.

[5] Volokh M., Peng G., Barrio J., Shalom M. (2019). Carbon Nitride Materials
for Water Splitting Photoelectrochemical Cells. Angew. Chem., Int. Ed..

[6] Ong W.-J., Tan L.-L., Ng Y. H., Yong S.-T., Chai S.-P. (2016). Graphitic
Carbon Nitride (g-C_3_N_4_)-Based Photocatalysts
for Artificial Photosynthesis and Environmental Remediation: Are We
a Step Closer To Achieving Sustainability?. Chem. Rev..

[7] Zhu J., Xiao P., Li H., Carabineiro S. A. C. (2014). Graphitic
Carbon Nitride: Synthesis, Properties, and Applications in Catalysis. ACS Appl. Mater. Interfaces.

[8] Miller T. S., Jorge A. B., Suter T. M., Sella A., Corà F., McMillan P. F. (2017). Carbon Nitrides:
Synthesis and Characterization of
a New Class of Functional Materials. Phys. Chem.
Chem. Phys..

[9] Barrio J., Volokh M., Shalom M. (2020). Polymeric Carbon Nitrides and Related
Metal-Free Materials for Energy and Environmental Applications. J. Mater. Chem. A.

[10] Wang X., Maeda K., Thomas A., Takanabe K., Xin G., Carlsson J. M., Domen K., Antonietti M. (2009). A Metal-Free
Polymeric Photocatalyst for Hydrogen Production from Water under Visible
Light. Nat. Mater..

[11] Shiraishi Y., Kanazawa S., Sugano Y., Tsukamoto D., Sakamoto H., Ichikawa S., Hirai T. (2014). Highly Selective
Production
of Hydrogen Peroxide on Graphitic Carbon Nitride (g-C_3_N_4_) Photocatalyst Activated by Visible Light. ACS Catal..

[12] Mazzanti S., Cao S., ten Brummelhuis K., Völkel A., Khamrai J., Sharapa D. I., Youk S., Heil T., Tarakina N. V., Strauss V., Ghosh I., König B., Oschatz M., Antonietti M., Savateev A. (2021). All-Organic Z-Scheme
Photoreduction of CO_2_ with Water as the Donor of Electrons
and Protons. Appl. Catal., B.

[13] Markushyna Y., Lamagni P., Catalano J., Lock N., Zhang G., Antonietti M., Savateev A. (2020). Advantages in Using Inexpensive CO_2_ To Favor
Photocatalytic Oxidation of Benzylamines. ACS
Catal..

[14] Uekert T., Bajada M. A., Schubert T., Pichler C. M., Reisner E. (2021). Scalable Photocatalyst
Panels for Photoreforming of Plastic, Biomass and Mixed Waste in Flow. ChemSusChem.

[15] Battula V. R., Mark G., Tashakory A., Mondal S., Volokh M., Shalom M. (2024). Binder-Free Carbon
Nitride Panels for Continuous-Flow
Photocatalysis. ACS Catal..

[16] Tashakory A., Battula V. R., Shalom M. (2025). Photocatalytic
Oxidation of Cyclohexane
in a Biphasic System Using Rapidly Synthesized Polymeric Carbon Nitride
Films. Photocatalysis.

[17] Abed B., Battula V. R., Volokh M., Garg D., Shmila T., Mark G., Tashakory A., Shames A. I., Shalom M. (2025). Selective
Toluene Oxidation Using Sulfur-Doped Polymeric Carbon Nitride Photocatalysts. Small.

[18] Rahaman M., Pulignani C., Miller M., Bhattacharjee S., Bin Mohamad Annuar A., Manuel R. R., Pereira I. A. C., Reisner E. (2025). Solar-Driven
Paired CO2 Reduction–Alcohol Oxidation Using Semiartificial
Suspension, Photocatalyst Sheet, and Photoelectrochemical Devices. J. Am. Chem. Soc..

[19] Garg D., Mark G., Battula V. R., Engel Y., Saini R. K., Shames A. I., Marichelvam T., Grave D. A., Volokh M., Shalom M. (2026). Vapor-Phase Synthesis
of Potassium-Doped Polymeric
Carbon Nitride Photocatalytic Panels. Small.

[20] Sun J., Schmidt B. V. K. J., Wang X., Shalom M. (2017). Self-Standing Carbon
Nitride-Based Hydrogels with High Photocatalytic Activity. ACS Appl. Mater. Interfaces.

[21] Lee W. H., Lee C. W., Cha G. D., Lee B.-H., Jeong J. H., Park H., Heo J., Bootharaju M. S., Sunwoo S.-H., Kim J. H., Ahn K. H., Kim D.-H., Hyeon T. (2023). Floatable Photocatalytic Hydrogel
Nanocomposites for Large-Scale
Solar Hydrogen Production. Nat. Nanotechnol..

[22] Xie Z., Yang W. (2025). A Robust Floating
Oxygen-Doped Graphitic Carbon Nitride Sheet for
Efficient Photocatalytic CO_2_ Conversion. Sep. Purif. Technol..

[23] Mazzanti S., Manfredi G., Barker A. J., Antonietti M., Savateev A., Giusto P. (2021). Carbon Nitride Thin Films as All-In-One
Technology for Photocatalysis. ACS Catal..

[24] Asadi A., Larimi A., Jiang Z., Naderifar A. (2022). Modeling and
Simulation of Photocatalytic CO_2_ Reduction into Methanol
in a Bubble Slurry Photoreactor. Chem. Eng.
Sci..

[25] Yang C., Li R., Zhang K. A. I., Lin W., Landfester K., Wang X. (2020). Heterogeneous Photoredox
Flow Chemistry for the Scalable Organosynthesis
of Fine Chemicals. Nat. Commun..

[26] Schimon D., Smitkova K., Stavarek P., Jaklova N., Vanluchene A., Dzik P., Homola T., Zazimal F., Kluson P. (2024). A Complex
Study of Photocatalytic Oxidation Pathways of Antibiotics with Graphitic
Carbon Nitride–The Way towards Continuous Flow Conditions. J. Environ. Chem. Eng..

[27] Chávez A. M., Torres-Pinto A., Álvarez P. M., Faria J. L., Silva C. G., Silva A. M. T. (2024). One-Pot
Synthesis and Immobilization of Urea-Derived
Graphitic Carbon Nitride onto Ceramic Foams for Visible-Light Photocatalytic
Wet Peroxide Oxidation in Water Treatment. Chem.
Eng. J..

[28] Jung H., Kim C., Yoo H.-W., You J., Kim J. S., Jamal A., Gereige I., Ager J. W., Jung H.-T. (2023). Continuous-Flow
Reactor with Superior Production Rate and Stability for CO_2_ Reduction Using Semiconductor Photocatalysts. Energy Environ. Sci..

[29] Pomberger A., Mo Y., Nandiwale K. Y., Schultz V. L., Duvadie R., Robinson R. I., Altinoglu E. I., Jensen K. F. (2019). A Continuous Stirred-Tank Reactor
(CSTR) Cascade for Handling Solid-Containing Photochemical Reactions. Org. Process Res. Dev..

[30] Peñas-Garzón M., Sampaio M. J., Manrique Y., Silva C. G., Faria J. L. (2023). Enhanced
Removal of Emerging Pollutants through Visible Light-Activated Carbon
Nitride Materials Immobilized over 3D Printed Structures. J. Environ. Chem. Eng..

[31] Lei L., Fan H., Jia Y., Wu X., Zhong Q., Wang W. (2022). Ultrafast
Charge-Transfer at Interfaces between 2D Graphitic Carbon Nitride
Thin Film and Carbon Fiber towards Enhanced Photocatalytic Hydrogen
Evolution. Appl. Surf. Sci..

[32] Swathi A. C., Sandhiya S. T., B S., Chandran M. (2024). Precursor Dependent
- Visible Light-Driven g-C_3_N_4_ Coated Polyurethane
Foam for Photocatalytic Applications. Chemosphere.

[33] Rogolino A., Linley S., Kwarteng P. K., Bonke S. A., Pulignani C., Reisner E. (2025). Floatable Carbon Nitride-Plastic
Composite for Paired
Photocatalysis at the Liquid-Liquid Interface. Chem.

[34] Shojaeimehr T., Tasbihi M., Acharjya A., Thomas A., Schomäcker R., Schwarze M. (2020). Impact of Operating
Conditions for the Continuous-Flow
Degradation of Diclofenac with Immobilized Carbon Nitride Photocatalysts. J. Photochem. Photobiol. A Chem..

[35] Tian S., Feng Y., Zheng Z., He Z. (2023). TiO_2_-Based
Photocatalytic Coatings on Glass Substrates for Environmental Applications. Coatings.

[36] Pedanekar R. S., Shaikh S. K., Rajpure K. Y. (2020). Thin Film
Photocatalysis for Environmental
Remediation: A Status Review. Curr. Appl. Phys..

[37] Wood D., Shaw S., Cawte T., Shanen E., Van Heyst B. (2020). An Overview
of Photocatalyst Immobilization Methods for Air Pollution Remediation. Chem. Eng. J..

[38] Thongsuriwong K., Amornpitoksuk P., Suwanboon S. (2013). Structure,
Morphology, Photocatalytic
and Antibacterial Activities of ZnO Thin Films Prepared by Sol–Gel
Dip-Coating Method. Adv. Powder Technol..

[39] Pham T.-T., Nguyen-Huy C., Lee H.-J., Nguyen-Phan T.-D., Son T. H., Kim C.-K., Shin E. W. (2015). Cu-Doped TiO_2_/Reduced Graphene Oxide Thin-Film Photocatalysts: Effect of
Cu Content upon Methylene Blue Removal in Water. Ceram. Int..

[40] Poongodi G., Kumar R. M., Jayavel R. (2015). Structural,
Optical and Visible Light
Photocatalytic Properties of Nanocrystalline Nd Doped ZnO Thin Films
Prepared by Spin Coating Method. Ceram. Int..

[41] Mohite S. V., Ganbavle V. V., Patil V. V., Rajpure K. Y. (2016). Photoelectrocatalytic
Degradation of Benzoic Acid Using Immobilized Tungsten Trioxide Photocatalyst. Mater. Chem. Phys..

[42] Lee H.-K., Fujiwara T., Okada T., Fukushima T., Lee S.-W. (2018). Fabrication of Visible-Light Responsive
N-Doped TiO_2_ Nanothin Films via a Top–down Sol–Gel
Deposition
Method Using NH_4_TiOF_3_ Single Crystals. Chem. Lett..

[43] Hu Y., Ping X., Zhang Y., Hao L., Liu T., Zhao Q., Lu Y., Liu J. (2021). A Nanotree-like WO_3_ Film with Adjustable Defect Concentration and Its Photocatalytic
Activity. Mater. Sci. Semicond. Process..

[44] Fouad K., Gar Alalm M., Bassyouni M., Saleh M. Y. (2020). A Novel Photocatalytic
Reactor for the Extended Reuse of W–TiO_2_ in the
Degradation of Sulfamethazine. Chemosphere.

[45] Fouad M., Gar Alalm M., El-Etriby H. K., Boffito D. C., Ookawara S., Ohno T., Fujii M. (2021). Visible-Light-Driven Photocatalytic
Disinfection of Raw Surface Waters (300–5000 CFU/ML) Using
Reusable Coated Ru/WO_3_/ZrO_2_. J. Hazard. Mater..

[46] Samy M., Ibrahim M. G., Gar Alalm M., Fujii M., Diab K. E., ElKady M. (2020). Innovative Photocatalytic
Reactor for the Degradation
of Chlorpyrifos Using a Coated Composite of ZrV_2_O_7_ and Graphene Nano-Platelets. Chem. Eng. J..

[47] Samy M., Ibrahim M. G., Gar Alalm M., Fujii M. (2020). MIL-53­(Al)/ZnO Coated
Plates with High Photocatalytic Activity for Extended Degradation
of Trimethoprim via Novel Photocatalytic Reactor. Sep. Purif. Technol..

[48] Alalm M. G., Djellabi R., Meroni D., Pirola C., Bianchi C. L., Boffito D. C. (2021). Toward Scaling-Up Photocatalytic
Process for Multiphase
Environmental Applications. Catalysts.

[49] Lee J. H., Kim S.-I., Park S.-M., Kang M. (2017). A P-n Heterojunction
NiS-Sensitized TiO_2_ Photocatalytic System for Efficient
Photoreduction of Carbon Dioxide to Methane. Ceram. Int..

[50] Fereidooni M., Márquez V., Paz C. V., Hensen E. J. M., Muravev V., Trongjitraksa P., Villanueva M. S., Praserthdam S., Praserthdam P. (2023). On the CO_2_ Photocatalytic Reduction over
Indium Tin Oxide (ITO) Ultra-Thin Films in Water Vapor: Experimental
and Theoretical Study. Fuel.

[51] Ravi P., Noh J. (2022). Photocatalytic Water
Splitting: How Far Away Are We from Being Able
to Industrially Produce Solar Hydrogen?. Molecules.

[52] Xiong A., Ma G., Maeda K., Takata T., Hisatomi T., Setoyama T., Kubota J., Domen K. (2014). Fabrication of Photocatalyst Panels
and the Factors Determining Their Activity for Water Splitting. Catal. Sci. Technol..

[53] Wang Q., Li Y., Hisatomi T., Nakabayashi M., Shibata N., Kubota J., Domen K. (2015). Z-Scheme Water
Splitting Using Particulate Semiconductors Immobilized
onto Metal Layers for Efficient Electron Relay. J. Catal..

[54] Wang Q., Hisatomi T., Suzuki Y., Pan Z., Seo J., Katayama M., Minegishi T., Nishiyama H., Takata T., Seki K., Kudo A., Yamada T., Domen K. (2017). Particulate Photocatalyst Sheets Based on Carbon Conductor Layer
for Efficient Z-Scheme Pure-Water Splitting at Ambient Pressure. J. Am. Chem. Soc..

[55] Wang Q., Hisatomi T., Jia Q., Tokudome H., Zhong M., Wang C., Pan Z., Takata T., Nakabayashi M., Shibata N., Li Y., Sharp I. D., Kudo A., Yamada T., Domen K. (2016). Scalable Water
Splitting on Particulate
Photocatalyst Sheets with a Solar-to-Hydrogen Energy Conversion Efficiency
Exceeding 1%. Nat. Mater..

[56] Sun S., Hisatomi T., Wang Q., Chen S., Ma G., Liu J., Nandy S., Minegishi T., Katayama M., Domen K. (2018). Efficient
Redox-Mediator-Free Z-Scheme Water Splitting Employing Oxysulfide
Photocatalysts under Visible Light. ACS Catal..

[57] Hisatomi T., Yamamoto T., Wang Q., Nakanishi T., Higashi T., Katayama M., Minegishi T., Domen K. (2018). Particulate Photocatalyst Sheets Based on Non-Oxide Semiconductor
Materials for Water Splitting under Visible Light Irradiation. Catal. Sci. Technol..

[58] Pan Z., Hisatomi T., Wang Q., Chen S., Nakabayashi M., Shibata N., Pan C., Takata T., Katayama M., Minegishi T., Kudo A., Domen K. (2016). Photocatalyst Sheets
Composed of Particulate LaMg_1/3_Ta_2/3_O_2_N and Mo-Doped BiVO_4_ for Z-Scheme Water Splitting under
Visible Light. ACS Catal..

[59] Pan Z., Hisatomi T., Wang Q., Chen S., Iwase A., Nakabayashi M., Shibata N., Takata T., Katayama M., Minegishi T., Kudo A., Domen K. (2016). Photoreduced Graphene
Oxide as a Conductive Binder to Improve the Water Splitting Activity
of Photocatalyst Sheets. Adv. Funct. Mater..

[60] Wang Q., Okunaka S., Tokudome H., Hisatomi T., Nakabayashi M., Shibata N., Yamada T., Domen K. (2018). Printable Photocatalyst
Sheets Incorporating a Transparent Conductive Mediator for Z-Scheme
Water Splitting. Joule.

[61] Goto Y., Hisatomi T., Wang Q., Higashi T., Ishikiriyama K., Maeda T., Sakata Y., Okunaka S., Tokudome H., Katayama M., Akiyama S., Nishiyama H., Inoue Y., Takewaki T., Setoyama T., Minegishi T., Takata T., Yamada T., Domen K. (2018). A Particulate
Photocatalyst
Water-Splitting Panel for Large-Scale Solar Hydrogen Generation. Joule.

[62] Wang Q., Domen K. (2020). Particulate Photocatalysts for Light-Driven
Water Splitting: Mechanisms,
Challenges, and Design Strategies. Chem. Rev..

[63] Nagatsuka K., Yoshino S., Yamaguchi Y., Kudo A. (2025). Facile Z-Scheme Photocatalyst
Sheets Consisting of Metal Sulfides and Poly-3,4-Ethylenedioxythiophene
of a Conductive Polymer for Visible-Light-Driven Water Splitting. Energy Fuels.

[64] Chandrappa S., Galbao S. J., Furube A., Murthy D. H. K. (2023). Extending the
Optical Absorption Limit of Graphitic Carbon Nitride Photocatalysts:
A Review. ACS Appl. Nano Mater..

[65] Lau V. W., Lotsch B. V. (2022). A Tour-Guide
through Carbon Nitride-Land: Structure-
and Dimensionality-Dependent Properties for Photo­(Electro)­Chemical
Energy Conversion and Storage. Adv. Energy Mater..

[66] Barrio J., Thomas A. (2025). Synthesis of Functional Materials Using N-Heterocyclic
Amines Beyond Melamine. Adv. Sci..

[67] Volokh M., Shalom M. (2023). Polymeric Carbon Nitride
as a Platform for Photoelectrochemical
Water-Splitting Cells. Ann. N.Y. Acad. Sci..

[68] Bian J., Li Q., Huang C., Li J., Guo Y., Zaw M., Zhang R.-Q. (2015). Thermal Vapor Condensation
of Uniform Graphitic Carbon
Nitride Films with Remarkable Photocurrent Density for Photoelectrochemical
Applications. Nano Energy.

[69] Liu Z., Wang C., Zhu Z., Lou Q., Shen C., Chen Y., Sun J., Ye Y., Zang J., Dong L., Shan C.-X. (2021). Wafer-Scale Growth
of Two-Dimensional
Graphitic Carbon Nitride Films. Matter.

[70] Lai B., Li T., Liu M., Cao H., Liu N., Zhao X., Luo X., Yu D., Zhao Y. (2026). Vapor-Solid Deposition and Condensation
of Graphitic Carbon Nitride Films and Their Charge Transport Behaviors
for Photoelectrochemical Applications. Appl.
Surf. Sci..

[71] Bian J., Li J., Kalytchuk S., Wang Y., Li Q., Lau T. C., Niehaus T. A., Rogach A. L., Zhang R.-Q. (2015). Efficient Emission
Facilitated by Multiple Energy Level Transitions in Uniform Graphitic
Carbon Nitride Films Deposited by Thermal Vapor Condensation. ChemPhysChem.

[72] Xu J., Brenner T. J. K., Chabanne L., Neher D., Antonietti M., Shalom M. (2014). Liquid-Based Growth of Polymeric Carbon Nitride Layers
and Their Use in a Mesostructured Polymer Solar Cell with Voc Exceeding
1 V. J. Am. Chem. Soc..

[73] Gu Q., Gong X., Jia Q., Liu J., Gao Z., Wang X., Long J., Xue C. (2017). Compact Carbon
Nitride
Based Copolymer Films with Controllable Thickness for Photoelectrochemical
Water Splitting. J. Mater. Chem. A.

[74] Jia C., Yang L., Zhang Y., Zhang X., Xiao K., Xu J., Liu J. (2020). Graphitic
Carbon Nitride Films: Emerging Paradigm for
Versatile Applications. ACS Appl. Mater. Interfaces.

[75] Xu J., Shalom M. (2016). Electrophoretic
Deposition of Carbon Nitride Layers
for Photoelectrochemical Applications. ACS Appl.
Mater. Interfaces.

[76] Wang Y., Zhao X., Tian Y., Wang Y., Jan A. K., Chen Y. (2017). Facile Electrospinning
Synthesis of Carbonized Polyvinylpyrrolidone
(PVP)/g-C_3_N_4_ Hybrid Films for Photoelectrochemical
Applications. Chem. - Eur. J..

[77] Arazoe H., Miyajima D., Akaike K., Araoka F., Sato E., Hikima T., Kawamoto M., Aida T. (2016). An Autonomous Actuator
Driven by Fluctuations in Ambient Humidity. Nat. Mater..

[78] Pulignani C., Mesa C. A., Hillman S. A. J., Uekert T., Giménez S., Durrant J. R., Reisner E. (2022). Rational Design of Carbon Nitride
Photoelectrodes with High Activity Toward Organic Oxidations. Angew. Chem., Int. Ed..

[79] Rieß J., Lublow M., Anders S., Tasbihi M., Acharjya A., Kailasam K., Thomas A., Schwarze M., Schomäcker R. (2019). XPS Studies
on Dispersed and Immobilised Carbon Nitrides Used for Dye Degradation. Photochem. Photobiol. Sci..

[80] Benedet M., Gallo A., Maccato C., Rizzi G. A., Barreca D., Lebedev O. I., Modin E., McGlynn R., Mariotti D., Gasparotto A. (2023). Controllable
Anchoring of Graphitic Carbon Nitride
on MnO_2_ Nanoarchitectures for Oxygen Evolution Electrocatalysis. ACS Appl. Mater. Interfaces.

[81] Abisdris L., Tzadikov J., Karjule N., Azoulay A., Volokh M., Shalom M. (2020). Electrophoretic Deposition
of Supramolecular Complexes
for the Formation of Carbon Nitride Films. Sustainable
Energy Fuels.

[82] Shalom M., Gimenez S., Schipper F., Herraiz-Cardona I., Bisquert J., Antonietti M. (2014). Controlled Carbon Nitride Growth
on Surfaces for Hydrogen Evolution Electrodes. Angew. Chem., Int. Ed..

[83] Peng G., Xing L., Barrio J., Volokh M., Shalom M. (2018). A General
Synthesis of Porous Carbon Nitride Films with Tunable Surface Area
and Photophysical Properties. Angew. Chem.,
Int. Ed..

[84] Tashakory A., Mondal S., Battula V. R., Mark G., Shmila T., Volokh M., Shalom M. (2024). Minute-Scale High-Temperature Synthesis
of Polymeric Carbon Nitride Photoanodes. Small
Struct..

[85] Xia J., Karjule N., Abisdris L., Volokh M., Shalom M. (2020). Controllable
Synthesis of Carbon Nitride Films with Type-II Heterojunction for
Efficient Photoelectrochemical Cells. Chem.
Mater..

[86] Zhao T., Zhou Q., Lv Y., Han D., Wu K., Zhao L., Shen Y., Liu S., Zhang Y. (2020). Ultrafast
Condensation of Carbon Nitride on Electrodes with Exceptional Boosted
Photocurrent and Electrochemiluminescence. Angew.
Chem., Int. Ed..

[87] Chubenko E. B., Maximov S. E., Bui C. D., Pham V. T., Borisenko V. E. (2023). Rapid Chemical
Vapor Deposition of Graphitic Carbon Nitride Films. Materialia.

[88] Liu J., Wang H., Chen Z. P., Moehwald H., Fiechter S., van de Krol R., Wen L., Jiang L., Antonietti M. (2015). Microcontact-Printing-Assisted
Access of Graphitic Carbon Nitride Films with Favorable Textures toward
Photoelectrochemical Application. Adv. Mater..

[89] Tashakory A., Karjule N., Abisdris L., Volokh M., Shalom M. (2021). Mediated Growth
of Carbon Nitride Films via Spray-Coated Seeding Layers for Photoelectrochemical
Applications. Adv. Sustainable Syst..

[90] Peng G., Albero J., Garcia H., Shalom M. (2018). A Water-Splitting Carbon
Nitride Photoelectrochemical Cell with Efficient Charge Separation
and Remarkably Low Onset Potential. Angew. Chem.,
Int. Ed..

[91] Qin J., Barrio J., Peng G., Tzadikov J., Abisdris L., Volokh M., Shalom M. (2020). Direct Growth of Uniform Carbon Nitride
Layers with Extended Optical Absorption towards Efficient Water-Splitting
Photoanodes. Nat. Commun..

[92] Shmila T., Mondal S., Barzilai S., Karjule N., Volokh M., Shalom M. (2023). Boron and Sodium Doping
of Polymeric Carbon Nitride
Photoanodes for Photoelectrochemical Water Splitting. Small.

[93] Karjule N., Barrio J., Xing L., Volokh M., Shalom M. (2020). Highly Efficient
Polymeric Carbon Nitride Photoanode with Excellent Electron Diffusion
Length and Hole Extraction Properties. Nano
Lett..

[94] Karjule N., Singh C., Barrio J., Tzadikov J., Liberman I., Volokh M., Palomares E., Hod I., Shalom M. (2021). Carbon Nitride-Based
Photoanode with Enhanced Photostability and Water Oxidation Kinetics. Adv. Funct. Mater..

[95] Karjule N., Phatake R. S., Barzilai S., Mondal B., Azoulay A., Shames A. I., Volokh M., Albero J., García H., Shalom M. (2022). Photoelectrochemical
Alcohols Oxidation over Polymeric
Carbon Nitride Photoanodes with Simultaneous H_2_ Production. J. Mater. Chem. A.

[96] Garg D., Shmila T., Mark G., Mondal S., Battula V. R., Volokh M., Shalom M. (2024). The Design
of Supramolecular Assemblies
with Metal Salt as Precursors Enables The Growth of Stable Polymeric
Carbon Nitride Photoanodes. Adv. Sustainable
Syst..

[97] Mondal S., Tashakory A., Mark G., Barzilai S., Pedersen A., Volokh M., Albero J., García H., Shalom M. (2025). Enhanced Activity and Stability of Polymeric Carbon
Nitride Photoanodes by Yttrium Incorporation. EES Catal..

[98] Mondal S., Naor T., Volokh M., Stone D., Albero J., Levi A., Vakahi A., García H., Banin U., Shalom M. (2024). NC Meets CN: Porous Photoanodes with
Polymeric Carbon Nitride/ZnSe Nanocrystal Heterojunctions for Photoelectrochemical
Applications. ACS Appl. Mater. Interfaces.

[99] Takata T., Jiang J., Sakata Y., Nakabayashi M., Shibata N., Nandal V., Seki K., Hisatomi T., Domen K. (2020). Photocatalytic Water Splitting with a Quantum Efficiency of Almost
Unity. Nature.

[100] Teng Z., Zhang Q., Yang H., Kato K., Yang W., Lu Y.-R., Liu S., Wang C., Yamakata A., Su C., Liu B., Ohno T. (2021). Author Correction:
Atomically Dispersed Antimony on Carbon Nitride for the Artificial
Photosynthesis of Hydrogen Peroxide. Nat. Catal..

[101] Teng Z., Zhang Q., Yang H., Kato K., Yang W., Lu Y.-R., Liu S., Wang C., Yamakata A., Su C., Liu B., Ohno T. (2021). Atomically
Dispersed Antimony on Carbon Nitride for the Artificial Photosynthesis
of Hydrogen Peroxide. Nat. Catal..

[102] Akaike K., Hosokai A., Nagashima H., Wei Q., Hosokai T. (2022). Chemical Reactions of Graphitic Carbon Nitride Films
with Glass Surfaces and Their Impact on Photocatalytic Activity. Phys. Chem. Chem. Phys..

[103] Bian J., Huang C., Zhang R.-Q. (2016). Graphitic
Carbon
Nitride Film: An Emerging Star for Catalytic and Optoelectronic Applications. ChemSusChem.

[104] Fina F., Callear S. K., Carins G. M., Irvine J. T. S. (2015). Structural
Investigation of Graphitic Carbon Nitride via XRD and Neutron Diffraction. Chem. Mater..

[105] Makuła P., Pacia M., Macyk W. (2018). How To Correctly
Determine
the Band Gap Energy of Modified Semiconductor Photocatalysts Based
on UV–Vis Spectra. J. Phys. Chem. Lett..

[106] Volokh M., Shalom M. (2021). Light on Peroxide. Nat. Catal..

[107] Kumar S., Battula V. R., Kailasam K. (2021). Single Molecular Precursors
for CxNy Materials- Blending of Carbon and Nitrogen beyond g-C_3_N_4_. Carbon.

[108] Vidyasagar D., Bhoyar T., Singh G., Vinu A. (2021). Recent Progress
in Polymorphs of Carbon Nitride: Synthesis, Properties, and Their
Applications. Macromol. Rapid Commun..

[109] Mondal S., Mark G., Abisdris L., Li J., Shmila T., Tzadikov J., Volokh M., Xing L., Shalom M. (2023). Developing Extended Visible Light Responsive Polymeric
Carbon Nitrides for Photocatalytic and Photoelectrocatalytic Applications. Mater. Horiz..

[110] Karjule N., Abisdris L., Azoulay A., Volokh M., Shalom M. (2022). Carbon-Doped Porous Polymeric Carbon
Nitride with Enhanced
Visible Light Photocatalytic and Photoelectrochemical Performance. Adv. Energy Sustainability Res..

[111] Miyata K., Shiraishi Y., Ichikawa S., Tanaka S., Hirai T. (2026). Porous Carbon Nitride
Photocatalysts Prepared by Calcination of Hydroxyl-Substituted
Melamine Derivatives. Chem. Commun..

[112] Barrio J., Barzilai S., Karjule N., Amo-Ochoa P., Zamora F., Shalom M. (2021). Synergistic Doping
and Surface Decoration
of Carbon Nitride Macrostructures by Single Crystal Design. ACS Appl. Energy Mater..

[113] Zhou Y., Zhang L., Liu J., Fan X., Wang B., Wang M., Ren W., Wang J., Li M., Shi J. (2015). Brand New P-Doped g-C3N4: Enhanced Photocatalytic Activity
for H_2_ Evolution and Rhodamine B Degradation under Visible
Light. J. Mater. Chem. A.

[114] Shah Y., Gilbertson L. M. (2025). Tuning
Reactive Species Production
of Graphitic Carbon Nitride via Oxygen Doping. ACS Sustainable Chem. Eng..

[115] Chen Z., Yan D., Wang X., Ding G., Wang Z., Xiao Y., Liu X., Wang P., Chen L., Shuai L., Liao G. (2025). Biochar-Tailored
Carbon
Nitride Enables Piezo-Photocatalytic H2O2 Production via Boosted Charge
Transport. ACS Catal..

[116] Li Q., Wang C., Yao H., He C., Guo C., Hu Y. (2025). Cocatalysts for Photocatalysis: Comprehensive
Insight into Interfacial
Charge Transfer Mechanism by Energy Band Theory. Coord. Chem. Rev..

[117] Akinaga Y., Kawawaki T., Kameko H., Yamazaki Y., Yamazaki K., Nakayasu Y., Kato K., Tanaka Y., Hanindriyo A. T., Takagi M., Shimazaki T., Tachikawa M., Yamakata A., Negishi Y. (2023). Metal Single-Atom Cocatalyst
on Carbon Nitride for the Photocatalytic Hydrogen Evolution Reaction:
Effects of Metal Species. Adv. Funct. Mater..

[118] Zhang X., Su H., Cui P., Cao Y., Teng Z., Zhang Q., Wang Y., Feng Y., Feng R., Hou J., Zhou X., Ma P., Hu H., Wang K., Wang C., Gan L., Zhao Y., Liu Q., Zhang T., Zheng K. (2023). Developing Ni Single-Atom Sites in
Carbon Nitride for Efficient Photocatalytic H2O2 Production. Nat. Commun..

[119] Cao M., Zhang Y., Feng H., Liu M., Liu D., Li Q. (2025). Synergistic Plasmonic and Molecular Engineering of
Carbon Nitride:
Breaking Photocatalytic Trade-Offs for Efficient Noble-Metal-Free
Solar CO_2_ Reduction. Adv. Funct.
Mater..

[120] Salati M., Mondal S., Volokh M., Albero J., García H., Gil-Sepulcre M., Shalom M., Llobet A. (2026). Efficient
and Robust Hybrid Molecular Photoanodes for Water Oxidation at Neutral
PH Based on Ru Complexes Covalently Bonded to Polymeric Carbon Nitride. Artif. Photosynth..

[121] Mondal S., Salati M., Nicaso M., Albero J., Segado-Centellas M., Volokh M., Bo C., García H., Gil-Sepulcre M., Llobet A., Shalom M. (2024). Supramolecular
Interaction
of a Molecular Catalyst with a Polymeric Carbon Nitride Photoanode
Enhances Photoelectrochemical Activity and Stability at Neutral PH. Chem. Sci..

[122] Wei Y., Chen L., Chen H., Cai L., Tan G., Qiu Y., Xiang Q., Chen G., Lau T.-C., Robert M. (2022). Highly Efficient
Photocatalytic Reduction of CO_2_ to CO by In Situ Formation
of a Hybrid Catalytic System Based on Molecular Iron Quaterpyridine
Covalently Linked to Carbon Nitride. Angew.
Chem., Int. Ed..

[123] Huang D., Yan X., Yan M., Zeng G., Zhou C., Wan J., Cheng M., Xue W. (2018). Graphitic
Carbon Nitride-Based Heterojunction Photoactive Nanocomposites: Applications
and Mechanism Insight. ACS Appl. Mater. Interfaces.

[124] Huang K.-H., Hou S.-S., Wu J.-J. (2021). Bridging Functional
Groups Governing the Charge Transfer Dynamic in an Amorphous Carbon
Nitride Allotropic Heterojunction toward Efficient Solar Hydrogen
Evolution. Sol. RRL.

[125] Peng G., Volokh M., Tzadikov J., Sun J., Shalom M. (2018). Carbon Nitride/Reduced Graphene Oxide Film with Enhanced
Electron Diffusion Length: An Efficient Photo-Electrochemical Cell
for Hydrogen Generation. Adv. Energy Mater..

[126] Chauhan D. K., Jain S., Battula V. R., Kailasam K. (2019). Organic Motif’s
Functionalization via Covalent Linkage in Carbon Nitride: An Exemplification
in Photocatalysis. Carbon.

[127] Peng X., Lei B., Tu Z., He M., Luo Q., Ding T., Wu S., Peng G. (2025). Synergy of
Triphase
Interface Engineering and Electronic Modulation of Carbon Nitride
Networks for Continuous-Flow Photosynthesis of H_2_O_2_. Adv. Funct. Mater..

[128] Li L., Cruz D., Savateev A., Zhang G., Antonietti M., Zhao Y. (2018). Photocatalytic Cyanation of Carbon Nitride Scaffolds: Tuning Band
Structure and Enhancing the Performance in Green Light Driven C–S
Bond Formation. Appl. Catal., B.

[129] Liu M., Wei C., Zhuzhang H., Zhou J., Pan Z., Lin W., Yu Z., Zhang G., Wang X. (2022). Fully Condensed Poly
(Triazine Imide) Crystals: Extended π-Conjugation and Structural
Defects for Overall Water Splitting. Angew.
Chem., Int. Ed..

[130] Cao Q., Kumru B., Antonietti M., Schmidt B. V. K. J. (2020). Graphitic
Carbon Nitride and Polymers: A Mutual Combination for Advanced Properties. Mater. Horiz..

[131] Karjule N., Phatake R., Volokh M., Hod I., Shalom M. (2019). Solution-Processable
Carbon Nitride Polymers for Photoelectrochemical
Applications. Small Methods.

[132] Linley S., Reisner E. (2023). Floating Carbon Nitride
Composites
for Practical Solar Reforming of Pre-Treated Wastes to Hydrogen Gas. Adv. Sci..

[133] Karak S., Liu Y., Annuar A. B. M., Reisner E. (2026). Covalent Organic
Framework and Carbon Nitride Composite for Scalable Solar Reforming. Adv. Mater..

[134] Zhang G., Lan Z.-A., Lin L., Lin S., Wang X. (2016). Overall Water Splitting by Pt/g-C_3_N_4_ Photocatalysts
without Using Sacrificial Agents. Chem. Sci..

[135] Zhang L., Hou S., Wang T., Liu S., Gao X., Wang C., Wang G. (2022). Recent Advances in Application of
Graphitic Carbon Nitride-Based Catalysts for Photocatalytic Nitrogen
Fixation. Small.

[136] Zheng Y., Zhang Y., Hang Z., Ouyang J., Chen Q., Guo X., Chen Z. (2026). Radical-Driven
Cooperative
Photocatalytic Coupling of Glucose Oxidation and H_2_O_2_ Production Using Ni-Doped Bi_2_WO_6_/CN
Heterojunction. J. Colloid Interface Sci..

[137] Zhang B., Kong L., Yan X., Zhang H., Wang Z., Xia S., Han Z., Xin Y., Ding A., Ma J., He X. (2025). Recent Progress in
Graphitic Carbon Nitride-Based Catalysts for Water Treatment: Contaminant
Elimination, Disinfection and Membrane Applications. Sep. Purif. Technol..

[138] Battula V. R., Mark G., Naeem M. S., Shah Y., Volokh M., Gilbertson L. M., López N., Shalom M. (2025). Light-Driven Chemical Cascade Reduces Barriers to Hydrogen
Production. J. Am. Chem. Soc..

[139] Meng S.-L., Zhang C., Li J.-H., Tung C.-H., Wu L.-Z. (2025). Photocatalytic
Cyclohexanone Oxime Synthesis from Ambient Air and
KA Oil. J. Am. Chem. Soc..

[140] An J., Zhao H., Jia Z., Zhao C., Cui C., Meng F., Sheng L., Wen M., Zheng Y., Xi T. (2025). Design and Engineering of Photocatalytic
Graphitic Carbon Nitride
for Environmental and Biological Disinfection. ACS EST Engg..

[141] Wei Q., Yang Y., Hou J., Liu H., Cao F., Zhao L. (2017). Direct Solar Photocatalytic Hydrogen Generation with
CPC Photoreactors:
System Development. Sol. Energy.

[142] Lin G., Huang M., Chen S., Li K., Han K., Liu M., Li Y., Chen W. (2025). Microstructure-Property
Relationships
in Cu/N-TiO_2_ Photocatalytic Concrete for Marine Applications:
Role of Preparation Strategies. J. Environ.
Chem. Eng..

[143] Alvarez-Fernandez A., Reid B., Fornerod M. J., Taylor A., Divitini G., Guldin S. (2020). Structural Characterization
of Mesoporous
Thin Film Architectures: A Tutorial Overview. ACS Appl. Mater. Interfaces.

[144] Andrei V., Wang Q., Uekert T., Bhattacharjee S., Reisner E. (2022). Solar Panel Technologies for Light-to-Chemical
Conversion. Acc. Chem. Res..

[145] Su Q., Sun J., Wang J., Yang Z., Cheng W., Zhang S. (2014). Urea-Derived Graphitic
Carbon Nitride as an Efficient Heterogeneous
Catalyst for CO_2_ Conversion into Cyclic Carbonates. Catal. Sci. Technol..

